# Increased fMRI connectivity upon chemogenetic inhibition of the mouse prefrontal cortex

**DOI:** 10.1038/s41467-022-28591-3

**Published:** 2022-02-25

**Authors:** Federico Rocchi, Carola Canella, Shahryar Noei, Daniel Gutierrez-Barragan, Ludovico Coletta, Alberto Galbusera, Alexia Stuefer, Stefano Vassanelli, Massimo Pasqualetti, Giuliano Iurilli, Stefano Panzeri, Alessandro Gozzi

**Affiliations:** 1 Functional Neuroimaging Laboratory, Center for Neuroscience and Cognitive systems, Istituto Italiano di Tecnologia, Rovereto, Italy; 2 Center for Mind and Brain Sciences, University of Trento, Rovereto, Italy; 3 Neural Computational Laboratory, Center for Neuroscience and Cognitive Systems, Istituto Italiano di Tecnologia, Rovereto, Italy; 4 Dept. of Biomedical Sciences and Padua Neuroscience Center, University of Padova, Padova, Italy; 5 Biology Department, University of Pisa, Pisa, Italy; 6 Systems Neurobiology Laboratory, Center for Neuroscience and Cognitive systems, Istituto Italiano di Tecnologia, Rovereto, Italy; 7 Department of Excellence for Neural Information Processing, Center for Molecular Neurobiology, University Medical Center Hamburg-Eppendorf, Hamburg, Germany

**Keywords:** Functional magnetic resonance imaging, Neural circuits

## Abstract

While shaped and constrained by axonal connections, fMRI-based functional connectivity reorganizes in response to varying interareal input or pathological perturbations. However, the causal contribution of regional brain activity to whole-brain fMRI network organization remains unclear. Here we combine neural manipulations, resting-state fMRI and in vivo electrophysiology to probe how inactivation of a cortical node causally affects brain-wide fMRI coupling in the mouse. We find that chronic inhibition of the medial prefrontal cortex (PFC) via overexpression of a potassium channel increases fMRI connectivity between the inhibited area and its direct thalamo-cortical targets. Acute chemogenetic inhibition of the PFC produces analogous patterns of fMRI overconnectivity. Using in vivo electrophysiology, we find that chemogenetic inhibition of the PFC enhances low frequency (0.1–4 Hz) oscillatory power via suppression of neural firing not phase-locked to slow rhythms, resulting in increased slow and δ band coherence between areas that exhibit fMRI overconnectivity. These results provide causal evidence that cortical inactivation can counterintuitively increase fMRI connectivity via enhanced, less-localized slow oscillatory processes.

## Abstract (web summary)

Pathological perturbation affects whole brain network activity. Here the authors show in mice that cortical inactivation unexpectedly results in increased fMRI connectivity between the manipulated regions and its direct axonal targets.

## Introduction

A rapidly expanding approach to understand the functional organization of brain networks is to map large-scale patterns of spontaneous activity via non-invasive neuroimaging. The ease and reproducibility of “resting state” fMRI (rsfMRI) have promoted the widespread use of this approach, leading to the observation that spontaneous fMRI activity is organized into highly coherent functional networks, defined by temporally correlated fluctuations in BOLD signal^1^. The non-invasive nature of rsfMRI has fueled the use of this method to map intrinsic brain network organization in the healthy human brain, as well as in psychiatric or neurological conditions, in which evidence of disrupted or aberrant rsfMRI functional coupling has been largely documented^1^. However, despite the growing popularity of rsfMRI, our knowledge of the underpinnings of brain-wide fMRI coupling remains very limited.

Multiple lines of evidence suggest that structural and rsfMRI-based connectivity are robustly related^1^. First, structural and functional connection strengths are correlated both at the whole-brain and mesoscopic scale^2–4^, and rsfMRI network topography closely recapitulates patterns of anatomical connectivity in several mammalian species^4–6^. Second, experimental resection of callosal connections^7^ or chemogenetic inactivation of the amygdala results in reduced rsfMRI connectivity with regions anatomically linked to the manipulated area^8^. Lastly, computational modeling corroborates a tight relationship between structural and functional connectivity, as synchronous rsfMRI fluctuations can be modeled by dynamical systems endowed with realistic anatomical connectivity patterns of long-range axonal interactions^9^. Accordingly, simulated axonal lesions in these models result in reduced functional coupling^10^.

These observations have prompted the widespread use of statistical dependencies in spontaneous fMRI signal as an index of interareal functional communication^1^. However, the neural mechanisms linking regional brain activity to large-scale rsfMRI network connectivity remain unclear. For example, growing experimental evidence suggests that, while tightly constrained by underlying anatomy, rsfMRI connectivity may only partially reflect direct interactions between areas, as widespread BOLD signal modulation might arise via subcortical connections, whether through the thalamus via long-range loops^11^, or as a result of diffuse neuromodulation mediated by brainstem nuclei^12,13^. This notion is epitomized by the observation of intact rsfMRI coupling among brain regions not directly structurally connected as in the case of acallosal humans, primates, and rodents^7,14,15^. Moreover, rsfMRI network topography can dynamically reconfigure in response to local perturbations^16^ or pathological processes^17^. In keeping with this, neurological disorders such as Parkinson’s disease, stroke, and Alzheimer’s disease have been often found to be associated with unexpectedly increased interareal rsfMRI connectivity despite the loss of cortical function characterizing these conditions^18,19^. Taken together, these observations point at a complex relationship between interareal brain activity and rsfMRI coupling, and call for a deeper investigation of the neural mechanisms underlying the reconfiguration of rsfMRI connectivity in response to varying interareal input or pathological perturbations.

Here we combine rsfMRI, neural and chemogenetic inhibition (chemo-fMRI^20^), and in vivo electrophysiology in the mouse to probe how inactivation of a cortical area causally affects rsfMRI coupling. Surprisingly, we find that chronic and acute inhibition of the medial prefrontal cortex (PFC), a core component of the mouse default mode network DMN^21^, can increase rsfMRI coupling with its thalamo-cortical targets. This effect is associated with decreased ϒ power in the suppressed region and robustly increased low frequency (0.1–4 Hz) electrophysiological coherence between functionally overconnected PFC targets. These findings reveal a highly dynamic, non-monotonic relationship between regional cortical activity and network-wide rsfMRI connectivity, and provide an interpretative framework for the observation of counterintuitively increased rsfMRI connectivity in pathological conditions characterized by impaired cortical function.

## Results

### rsfMRI overconnectivity upon chronic inactivation of the mouse prefrontal cortex

**Fig. 1:**
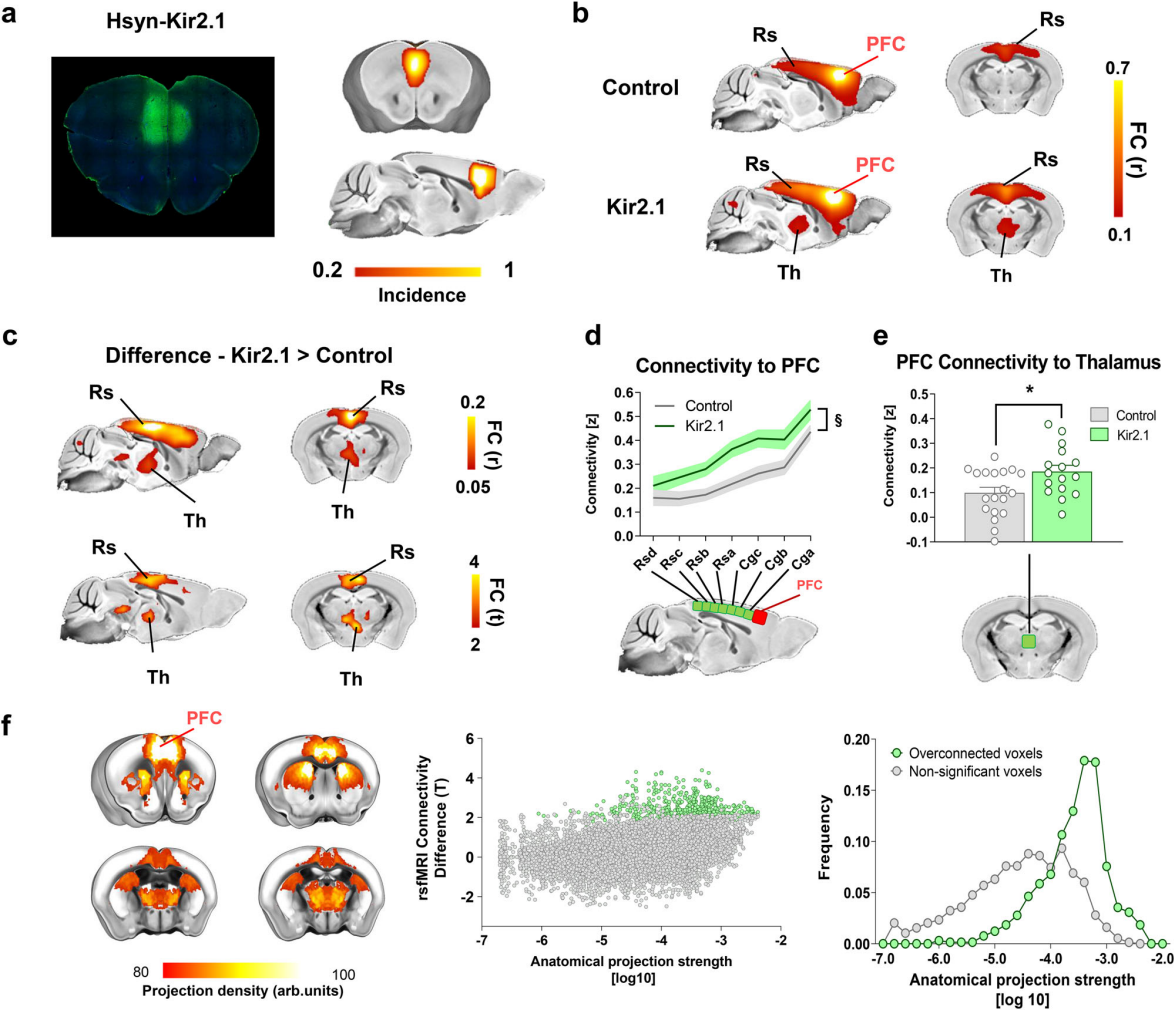
Chronic inhibition of the mouse PFC results in rsfMRI overconnectivity. **a** Viral expression localization. The potassium channel Kir2.1 (*n* = 16) or GFP (control, *n* = 19) were transduced bilaterally into the PFC of adult male mice. Left: representative histology sample showed Kir2.1 (green). Right: heatmaps illustrate a qualitative regional assessment of viral expression across subjects. **b** Seed-based connectivity mapping of the PFC in GFP (control), and Kir2.1-transduced subjects. **c** Corresponding group difference maps. Area exhibiting significantly increased rsfMRI connectivity in Kir2.1 expressing mice are depicted in red-yellow (r and T stat difference map). **d** Antero-posterior profiling of rsfMRI connectivity of the PFC within the midline axis of the mouse DMN (§*p* = 0.014, two-way ANOVA with repeated measurements, genotype effect, *n* = 16 and *n* = 19 Kir2.1 or GFP-expressing mice, respectively). **e** Fronto-thalamic rsfMRI overconnectivity in Kir2.1 expressing mice (*p = 0.014, two-sided *t* test, *n* = 16 and *n* = 19 Kir2.1 or GFP-expressing mice, respectively). Data in **e** and **f** are presented as mean values ± SEM. **f** Regions exhibiting rsfMRI overconnectivity in Kir2.1 mice are robustly innervated by the PFC. Left: axonal projections from the PFC (top 20% strongest connections). Middle: scatter plot illustrating intergroup differences in rsfMRI connectivity as a function of PFC structural connectivity strength. Green dots indicate significantly functionally overconnected voxels. Right: Distribution of overconnected voxels as a function of axonal connectivity strength . FC functional connectivity, DMN Default Mode Network, Cg cingulate cortex, PFC prefrontal cortex, Rs retrosplenial, Th thalamus. Source data are provided as a Source Data file.

The robust structural foundations of rsfMRI connectivity suggest that neural inhibition of a network node would result in diminished functional coupling with regions receiving direct axonal projections from the inactivated region^8,10,22^. To test this prediction and more broadly investigate how rsfMRI dynamically reconfigures in response to local neural suppression, we carried out rsfMRI measurements in a cohort of mice in which neuronal activity in PFC was chronically inhibited via bilateral viral transduction of the inward rectifying potassium channel Kir2.1 (Fig. 1a). Our interest in the PFC was motivated by its translational relevance as a key component of the mouse default mode network (DMN), a major phylogenetically conserved rsfMRI network that in rodents is composed of three hubs, namely the PFC, the retrosplenial cortex, and the medial thalamus^4^. Prior research in awake animals has shown that virally‐mediated Kir2.1 expression results in a reduction of both evoked and spontaneous neuronal excitability lasting several weeks^23,24^. In keeping with this, in vivo electrophysiological recordings in the PFC of mice unilaterally transfected with Kir2.1 revealed a robust reduction of spontaneous firing rate in the targeted cortical area with respect to its contralateral control regions (*N* = 4, paired *t* test, *p* = 0.002, Fig. S1).

We next compared the patterns of rsfMRI connectivity in Kir2.1 and GFP-transduced control littermates by imaging Kir2.1 transfected mice four weeks after viral injections (Fig. 1a). Consistent with previous investigations^25,26^, seed-based probing revealed significant long-range correlation between the PFC and thalamo-cortical components of the mouse DMN in both cohorts (Fig. 1b). Surprisingly, between-group comparisons revealed foci of significantly increased rsfMRI connectivity in the posterior cingulate/retrosplenial cortex and centromedial thalamic regions of Kir2.1 transfected mice (*t* test, *p* < 0.05, *t* > 2.03, FWE cluster-corrected, *p* < 0.05; Fig. 1c). Regional quantifications of DMN connectivity via multiple prefrontal-DMN seeds corroborated these findings, revealing increased rsfMRI synchronization along the entire midline extension of this network (two-way repeated measures ANOVA, *F*_1,33_ = 6.93; *p* = 0.013; Fig. 1d) and its centromedial thalamic targets (*t* test, *t*_33_ = 2.589, *p* = 0.014; Fig. 1e). Voxel-wise mapping did not reveal any foci of reduced functional connectivity with the PFC (*t* > 2.03, FWE cluster-corrected, *p* < 0.05). Importantly, all the thalamic and cortical regions showing increased rsfMRI connectivity in Kir2.1 mice are characterized by high axonal projection density from the PFC, as seen by comparing the magnitude of inter-group rsfMRI connectivity differences with incoming axonal connectivity strength inferred from a voxel-model of the mouse brain connectome^4^ (Fig. 1f, Wilcoxon rank-sum test, *p* < 0.0001). Interestingly, the direction and anatomical location of DMN rsfMRI overconnectivity was not altered by global fMRI signal regression (Fig. S2), with the exception of thalamic areas, in which the connectivity difference between groups was attenuated. Together, these findings reveal that chronic inhibition of neural activity in the PFC may counterintuitively increase rsfMRI functional connectivity between long-range thalamo-cortical targets of the mouse DMN.

### rsfMRI overconnectivity upon acute chemogenetic inactivation of the mouse prefrontal cortex

To corroborate the specificity of Kir2.1 findings and obtain mechanistic insight into the neural correlates of the observed fMRI overconnectivity, we designed a new set of experiments in which DREADD-based chemogenetics was employed to induce a time-controlled, acute inhibition of PFC activity during rsfMRI scanning. It should be noted that the same manipulations we hereafter refer to as “chemogenetic inhibition/inactivation”, are often termed and interpreted as “chemogenetic silencing” in neuro-behavioral neuroscience^27^.

**Fig. 2:**
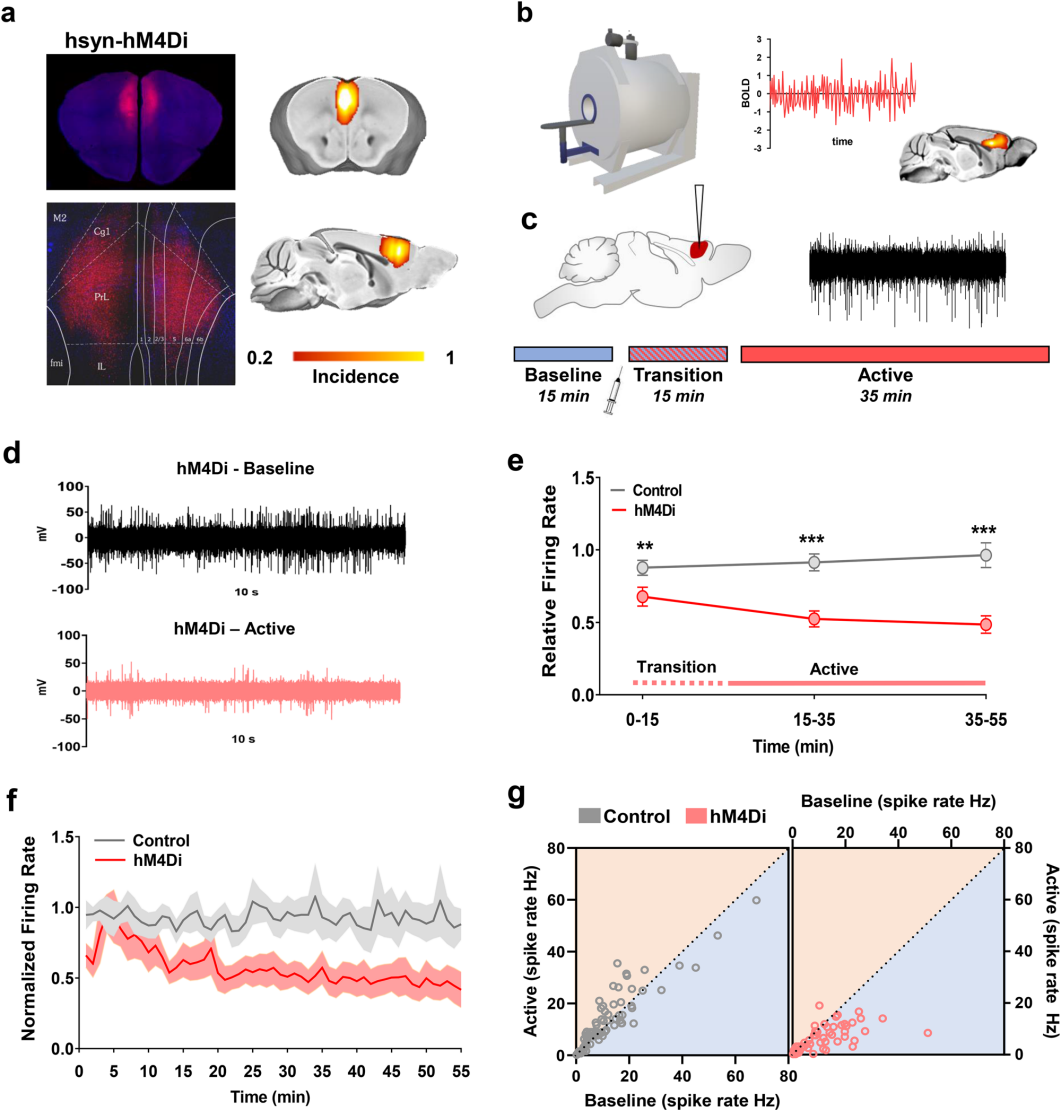
Chemogenetic inhibition of neural firing in the PFC. **a** Experimental design of chemo-fMRI experiments. AAV8-hSyn-hM4Di (*n* = 15) or AAV8-hSyn-GFP (control, *n* = 19) were bilaterally injected into the PFC of wild type. Left: representative histology sample showed hM4Di (red) expression. Right: heatmaps illustrate a qualitative regional assessment of viral expression across subjects. **b** Mice underwent chemo-fMRI scanning or **c** electrophysiological recordings to probe effectiveness of chemogenetic manipulations. A reference acquisition timeline is reported to depict timeseries binning into a 15-min pre-CNO reference baseline, a drug equilibration window (15 min, transition), and a 35-min CNO active time window (active). **d** Representative raw traces collected before and after CNO injection in representative recordings site of a hM4Di-expressing mouse. **e**, **f** Reduced firing rate in hM4Di-expressing mice (*n* = 5) compared to GFP-transduced controls (*n* = 5, two-sided Wilcoxon rank-sum tests, FDR corrected **q < 0.01, ***q < 0.001). Data are presented as mean values ± SEM. **g** Scatterplot comparing the firing rate of individual PFC recording channels during baseline conditions (*x* axis) and the active phase (*y* axis) in control and DREADD-expressing animals (two-sided Wilcoxon rank-sum test FDR corrected, **q < 0.01, ***q < 0.001). Source data are provided as a Source Data file.

An overview of experimental procedures is provided in Fig. 2. To enable remote inhibition of fronto-cortical activity, we bilaterally transfected the PFC with the inhibitory hM4Di DREADD using a pan-neuronal promoter (Fig. 2a), a strategy widely used to regionally inhibit excitatory neural function in behavioral studies^27^. In keeping with previous investigations, the use of a high titer viral suspension resulted in reliable and homogeneous transduction of neurons across cortical layers^28^. Three weeks after viral injection, control (GFP-transfected) and hM4Di-expressing animals underwent rsfMRI scanning or electrophysiological recordings before and after intravenous injection of the DREADD activator clozapine-N-oxide (CNO). To account for the relatively slow pharmacokinetic profile of CNO in the rodent brain^29,30^, both imaging and electrophysiological recordings were split into a pre-CNO injection baseline, a transitory (0–15 min) drug-equilibration period, and an active time window (15–50 min post CNO injection) to which all our analyses refer to, unless otherwise specified (Fig. 2b, c).

To test the efficacy of chemogenetic inhibition, we first performed a set of electrophysiological recordings in the PFC of hM4Di- or GFP-transduced control animals prior to and after CNO administration, under the same experimental conditions used in rsfMRI imaging (Fig. 2d, e). Baseline electrophysiological traces revealed the presence of appreciable spontaneous multi-unit activity (MUA) in the PFC of both groups (mean firing rate 15.0 ± 2.2 spikes/s in hM4Di-expressing, and 14.1 ± 3.8 in GFP-transduced mice, *n* = 5 each group, *p* = 0.851, *t* test). As expected, CNO administration robustly inhibited firing rate in hM4Di expressing mice, but not in control subjects (Fig. 2e, f, *p* < 0.01 FDR corrected, *t* test). DREADD-induced PFC inhibition was characterized by a gradual decrease of neural firing upon CNO administration, reaching a steady-state ~10–15 min after the intravenous bolus (Fig. 2f).

Prompted by recent work suggesting that DREADDs may alter or disrupt, rather than complete silence, neuronal activity in vivo^31^, we next examined more in detail the firing patterns of individual electrode sites in MUA recordings. For each group, we compared the firing rates of each MUA site under basal conditions with those obtained in the same recording site upon CNO administration. A scatter plot of spike rate across paired conditions revealed that, during the active phase, the vast majority of MUA sites in DREADD-expressing mice showed a marked decrease in firing rate, with virtually no site showing any appreciable decrease in firing in control animals (Fig. 2g). Given that MUA is strongly biased by the spiking activity of pyramidal neurons^32^, these analyses suggest that our chemogenetic manipulations produce a general decrease in excitatory firing.

**Fig. 3:**
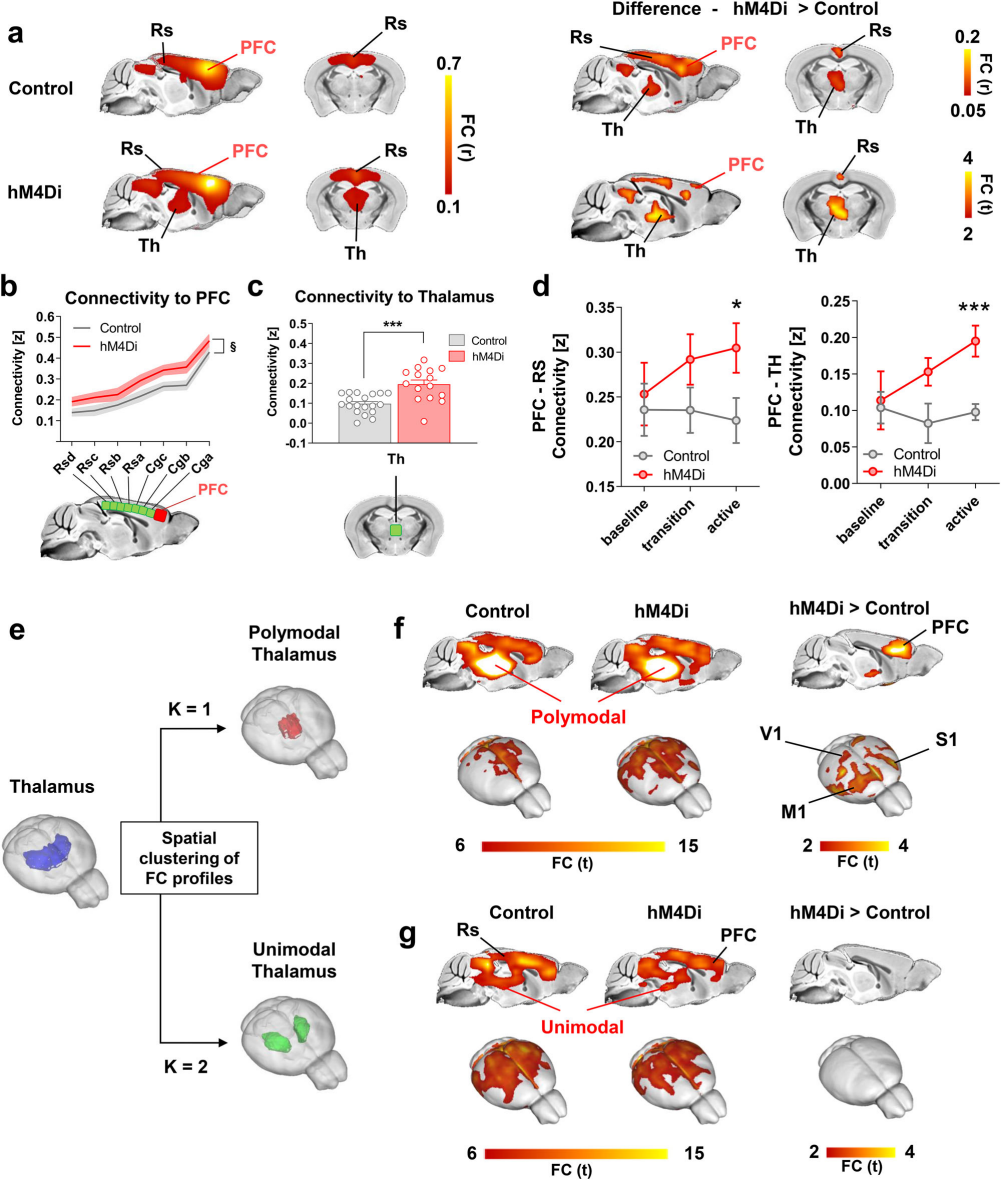
Chemogenetic inhibition of the mouse PFC results in rsfMRI overconnectivity. **a** Seed-based connectivity of the PFC and between group difference map revealed rsfMRI over-connectivity in the DMN of hM4Di expressing mice during the active phase. **b** Antero-posterior profiling of rsfMRI connectivity of the PFC along the midline axis of the mouse DMN in the two cohorts (§*p* = 0.106, two-way ANOVA repeated measurements, genotype effect, *n* = 15 and *n* = 19 control or hM4Di-expressing animals, respectively). **c** Thalamo-cortical rsfMRI hyper synchronization in hM4Di expressing mice and **d** prefrontal-retrosplenial and prefrontal-thalamic connectivity timecourse (*p = 0.039, ***p < 0.001, two-sided *t* test, *n* = 15 and *n* = 19 hM4Di or GFP-expressing mice, respectively.) Data in **b**–**d** are presented as mean values ±SEM. **e** k-means clustering of PFC-thalamic rsfMRI connectivity profiles (thalamus, blue; polymodal thalamus, red; unimodal thalamus, green) in Control (*n* = 19) and hM4Di (*n* = 15) animals. **f**, **g** Seed connectivity of sub-thalamic partitions (**f** polymodal thalamus; **g** unimodal thalamus) and corresponding between group difference maps. FC functional connectivity, Cg cingulate cortex, M1 motor cortex, S1 sensory cortex, PFC prefrontal cortex, Rs retrosplenial cortex, Th thalamus, V1 visual cortex. Source data are provided as a Source Data file.

To probe whether acute chemogenetic inhibition of the PFC would produce rsfMRI overconnectivity as observed with Kir2.1, we next compared rsfMRI connectivity patterns in hM4Di transfected and control mice upon acute CNO administration (active phase, Fig. 2). Recapitulating the results of chronic PFC inhibition, voxel-wise mapping revealed foci of significantly increased rsfMRI connectivity in the posterior cingulate/retrosplenial cortices and midline thalamic regions of DREADD-expressing mice (*t* test, *p* < 0.05, *t* > 2.03, FWE cluster-corrected, *p* < 0.05; Fig. 3a). Regional quantifications corroborated the presence of rsfMRI overconnectivity along the cingulate and retrosplenial axis of the DMN, and between the PFC and medio-dorsal thalamic areas (two-way repeated measures ANOVA, *F*_1,32_ = 6.58; *p* = 0.016; *t* test *t*_32_ = 4.30, *p* = 0.001, respectively; Fig. 3b, c), a set of regions characterized by dense incoming projections from the PFC (Fig. S3, Wilcoxon rank-sum test, *p* < 0.0001). Notably, the direction and the anatomical location of the observed rsfMRI overconnectivity was regionally unaltered by global fMRI signal regression (Fig. S2), arguing against an unspecific contribution of arousal related global dynamics or global fMRI co-activation to the mapped changes^33,34^.

Baseline PFC connectivity in these areas was comparable across groups (voxel-wise mapping, Z > 2.03 cluster corrected, PFC-Cingulate, two-way ANOVA, *F*_1,32_ = 0.48, *p* = 0.490, Thalamo-PFC, *t* test, *t*_32_ = 0.23, *p* = 0.817), and overconnectivity gradually emerged in the hM4Di cohort after CNO administration, peaking during the DREADD active time-window (PFC-Rs: *T*_32_ = 2.158, *p* = 0.038, PFC-Th: *T*_32_ = 4.301, *p* = 0.0001, *t* test, Fig. 3d). Moreover, no intergroup differences were observed in the estimated characteristic hemodynamic response function in this area (kernel height *p* > 0.604; time-to-peak *p* > 0.123, full-width-at-half-peak *p* > 0.376, *t* test) nor were between-group differences in arterial blood pressure (*p* > 0.7, *t* test) or blood gas levels observed (P_a_CO_2_ *p* = 0.489; P_a_O_2_ *p* = 0.223, *t* test). These control measurements rule out major spurious vascular or hemodynamic contributions and corroborate the specificity of the mapped changes. Importantly, a replication of our chemo-fMRI study in a new set of animals imaged using a combination of medetomidine and low-dose isoflurane^35,36^ revealed increased rsfMRI coupling between PFC and thalamic and retrosplenial areas (Fig. S4), recapitulating our findings in halothane-anesthetized mice. This result suggests that the observed hyperconnectivity does not reflect pharmacological interaction of DREADDs with the specific anesthetic used, but represents a more general phenomenon that extends to other sedatives and anesthetic conditions. More broadly, our chemo-fMRI results show that acute inactivation of PFC activity results in a pattern of DMN overconnectivity closely recapitulating that observed with chronic Kir2.1-mediated neural inhibition, suggesting that the ensuing overconnectivity is not manipulation-specific, nor the indirect consequence of homeostatic reactivity to protracted neural suppression.

### Chemogenetic inhibition of the prefrontal cortex leads to thalamo-cortical rsfMRI overconnectivity

Topographical mapping of rsfMRI connectivity upon chemogenetic inhibition of the PFC revealed foci of hyperconnectivity in polymodal medio-dorsal and centro-medial areas of the thalamus, a set of higher-order nuclei densely innervated by the mouse PFC^37^. We thus probed the rsfMRI connectivity of the thalamus to assess whether the observed foci of thalamic overconnectivity could underlie or involve the engagement of additional brain regions outside the DMN. To obtain a regionally unbiased identification of polymodal (i.e. densely-PFC projecting) versus more unimodal lateral portions of the thalamus, we used k-means (*k* = 1–3) and hierarchical clustering to partition thalamic voxels based on their whole-brain rsfMRI connectivity profile^38^. Consistent with the neuroanatomical organization of this region, our approach revealed two segregable thalamic sub-territories, one encompassing its centromedial and anterodorsal (PFC-innervated) polymodal components, and the second encompassing more lateral (unimodal/sensory) areas (Fig. 3e). This anatomical segregation is also of potential mechanistic interest, as polymodal thalamic areas have been recently shown to serve as key generators and cortical propagators of slow neural rhythms (e.g. δ) relevant to rsfMRI coupling^11,39–41^.

Interestingly, seed-based probing of the unimodal thalamus did not reveal significant rsfMRI connectivity differences between hM4Di-expressing and control animals (Fig. 3g), whereas seed-based probing of the polymodal thalamus revealed in hM4Di expressing-mice a widespread pattern of cortical overconnectivity, exceeding the boundaries of the PFC to encompass motor and somatosensory territories, including the retrosplenial cortex (Fig. 3f, *t* test, *p* < 0.05, *t* > 2.03, FWE cluster-corrected, *p* < 0.05, Fig. S5). To probe the network specificity of these effects, we next mapped whole-brain rsfMRI connectivity in hM4Di and control animals using a whole-brain parcellation scheme (Fig. S6). This analysis revealed the presence of significant clusters of rsfMRI overconnectivity only in regions of the DMN and in polymodal thalamic areas of DREADD-expressing animals, the latter areas exhibiting overconnectivity with larger cortical regions. No meaningful clusters of rsfMRI over- or under-connectivity were otherwise observed in any of the areas probed. These results show that, upon acute suppression of fronto-cortical activity, PFC-innervated polymodal thalamic regions, but not unimodal areas, exhibit over-synchronous rsfMRI coupling with large cortical areas.

### Chemogenetic inactivation of the prefrontal cortex inhibits firing not locked to slow rhythms, resulting in increased slow oscillatory power

**Fig. 4:**
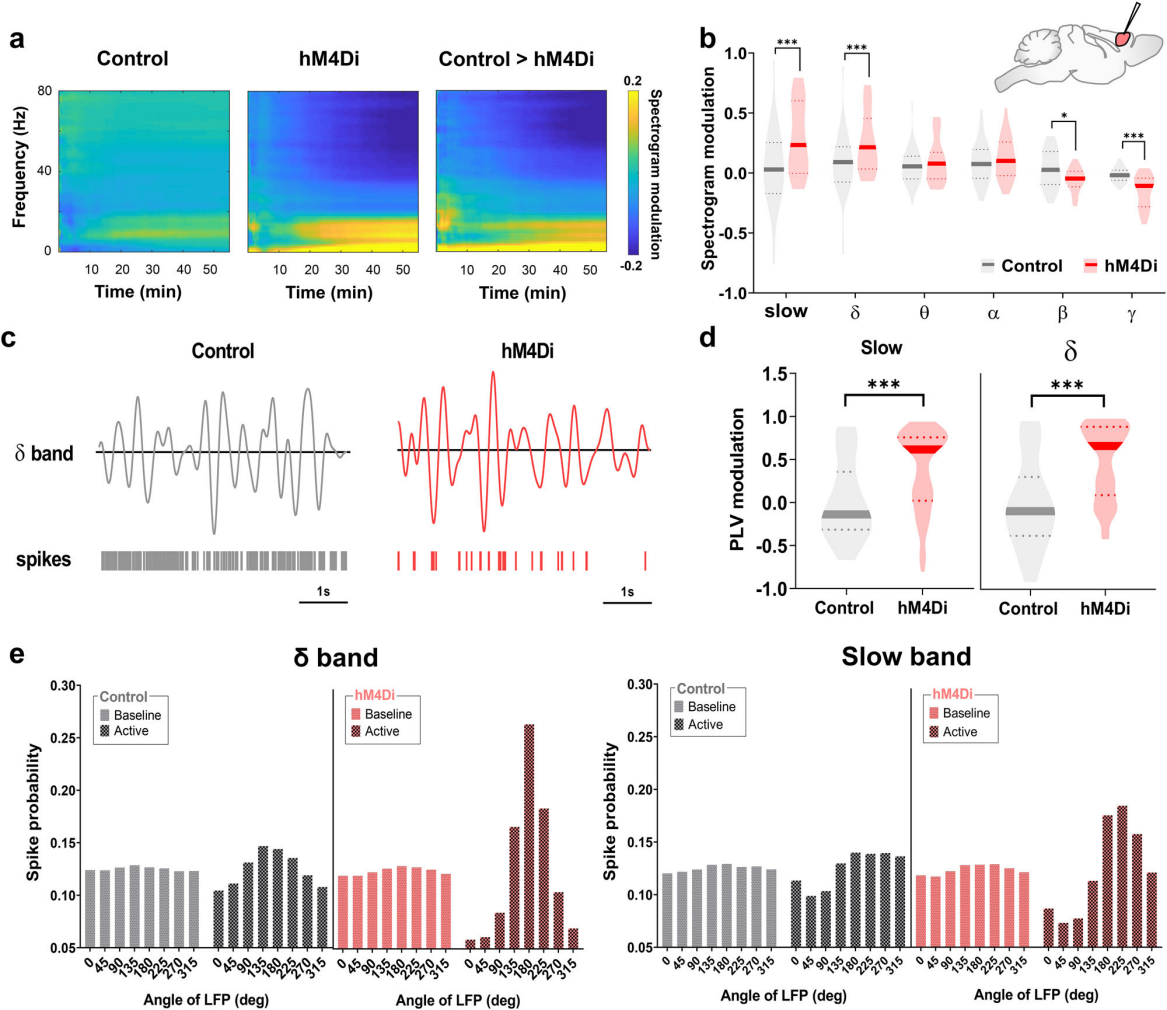
Chemogenetic inhibition of the PFC reduces ƴ activity but increases slow oscillatory power. **a** Mean post-injection spectrogram in control (left), hM4Di-expressing animals (center), and mean between group difference (right). **b** Quantification of band-specific power spectrum changes upon CNO injection in both groups (*q < 0.05, ***q < 0.001, two-sided Wilcoxon rank-sum tests followed by FDR correction, for *n* = 50 and *n* = 60 statistically independent recordings from *n* = 5 hM4Di and *n* = 5 control mice, respectively). **c** Example traces of band-passed δ-band LFPs and corresponding spiking activity (right) from a representative PFC recording channel during active phase in control and DREADD-expressing mice. Note the presence of greatly reduced, but more phase-locked firing in animals expressing hM4Di channel. **d** Violin plots depicting PLV of PFC spikes to slow and δ bands (***q < 0.001, two-sided Wilcoxon rank-sum tests followed by FDR correction for *n* = 79 and *n* = 80 statistically independent recordings from *n* = 5 hM4Di and *n* = 5 control mice, respectively). **e** Probability of firing (all sessions and datapoints) as a function of the phase angle of δ (bottom) and slow (top) bands. Phase conventions are such that 0 and 180 deg represent the peak and through of LFP, respectively. (Violin plots: thick lines represent median, dashed lines indicate 25th and 75th percentile, respectively). PLV phase-locking value. Source data are provided as a Source Data file.

To obtain insight into the neural rhythms underlying the observed rsfMRI overconnectivity, we next analyzed local field potential (LFP) traces obtained from the same set of PFC electrophysiological recordings analyzed in Fig. 2. Consistent with the observed firing rate reduction, LFP spectrograms from hM4Di-transduced animals revealed an overall shift of LFP power towards lower frequencies, with a robust decrease in β- and ƴ-band power after CNO administration with respect to CNO-treated controls (*F*_1,22_ = 239.4, *p* < 0.1 and *p* < 0.001, respectively, Fig. 4a, b). Notably, the LFP power reduction in these frequency bands was also accompanied by a prominent increase in δ and slow-band (defined here as 0.1–1 Hz) LFP power in DREADD-expressing subjects (Fig. 4b, slow *p* < 0.001, δ *p* < 0.001, θ *p* = 0.779; α *p* = 0.340; β *p* = 0.017; ƴ *p* < 0.001; two-sided Wilcoxon rank-sum tests, FDR corrected). Given that higher LFP frequencies typically reflect local neural interactions, while lower LFP frequencies are instead associated with larger-scale phenomena^32,42^, these results suggest that chemogenetic inhibition of PFC suppresses local, rather than global, neural processes.

To investigate the relationship between DREADD-induced reduction in firing and the concomitant increase in local slow and δ oscillatory power, we next performed phase locking analyses of MUA firing in the PFC. Interestingly, our investigations revealed that in DREADD-expressing animals, the fewer residual spikes recorded after CNO injection exhibited strong phase locking to ongoing slow and δ LFP oscillations. Such strong phase locking was not observed in control animals or in baseline recordings, where spike distributions were instead more equally spread across δ and slow oscillation phases (Fig. 4c–e). These observations suggest that chemogenetic inhibition of the PFC leads to a selective reduction of firing not locked to ongoing slow rhythms, resulting in a concomitant increased phase locking of residual firing to high-excitability phases of ongoing low-frequency oscillations. The resulting spiking activity is therefore on average greatly reduced, but also considerably more periodic and phase-locked to underlying low-frequency oscillatory rhythms.

To better illustrate the relationship between increased oscillation power and enhanced spike locking in the slow and δ bands, we performed a simple simulation. Specifically, we first simulated spike trains to have weak phase locking to a slow rhythm, with spikes happening almost equally across phase angles of the oscillatory cycle as observed in control conditions. To mimic DREADD-induced effects, we next selectively removed spikes emitted at the non-preferred phases of slow oscillatory activity as observed in our recordings. To mimic DREADD-induced effects, we next selectively removed spikes emitted at the non-preferred phases of slow oscillatory activity as observed in our recordings. In keeping with our experimental results, this simple simulation showed that the removal of “asynchronous” off-preferred-phase firing reduces power of high-frequency spiking activity, while simultaneously increasing the power of the low-frequency rhythm residual spikes are entrained to (Fig. S7). This suggests that DREADD-induced increase in slow LFP power does not necessarily reflect the generation of a new, “artificial” rhythm, but can be simply explained by the emergence of increased phase locking to existing slow oscillatory activity.

Finally, to further probe the mechanistic specificity of our findings, we measured rsfMRI connectivity profiles produced by cell-type-specific manipulations designed to increase local PFC firing. We found that chemogenetic inhibition of fast-spiking Parvalbumin (PV) GABAergic cells or hM3Dq-based excitation of pyramidal (CamkII-expressing) neurons both produced PFC underconnectivity with cortical terminals of the DMN (Fig. S8). Notably, both manipulations produced neural signatures characterized by increased local firing rate and a robust shift of LFP power towards higher frequencies, effectively reversing the corresponding electrophysiological signature observed in our DREADD inhibition studies (Fig. 4). These effects were far more prominent in mice undergoing DREADD-based stimulation of pyramidal neurons, a manipulation that was also associated with largely suppressed slow and δ-band LFP activity. Taken together, these new investigations suggest that the observed functional hyperconnectivity does not reflect an increased excitatory/inhibitory ratio in the PFC, or an unspecific functional inhibition of GABAergic neurons.

### Chemogenetic inhibition of the prefrontal cortex increases interareal slow oscillatory coherence

**Fig. 5:**
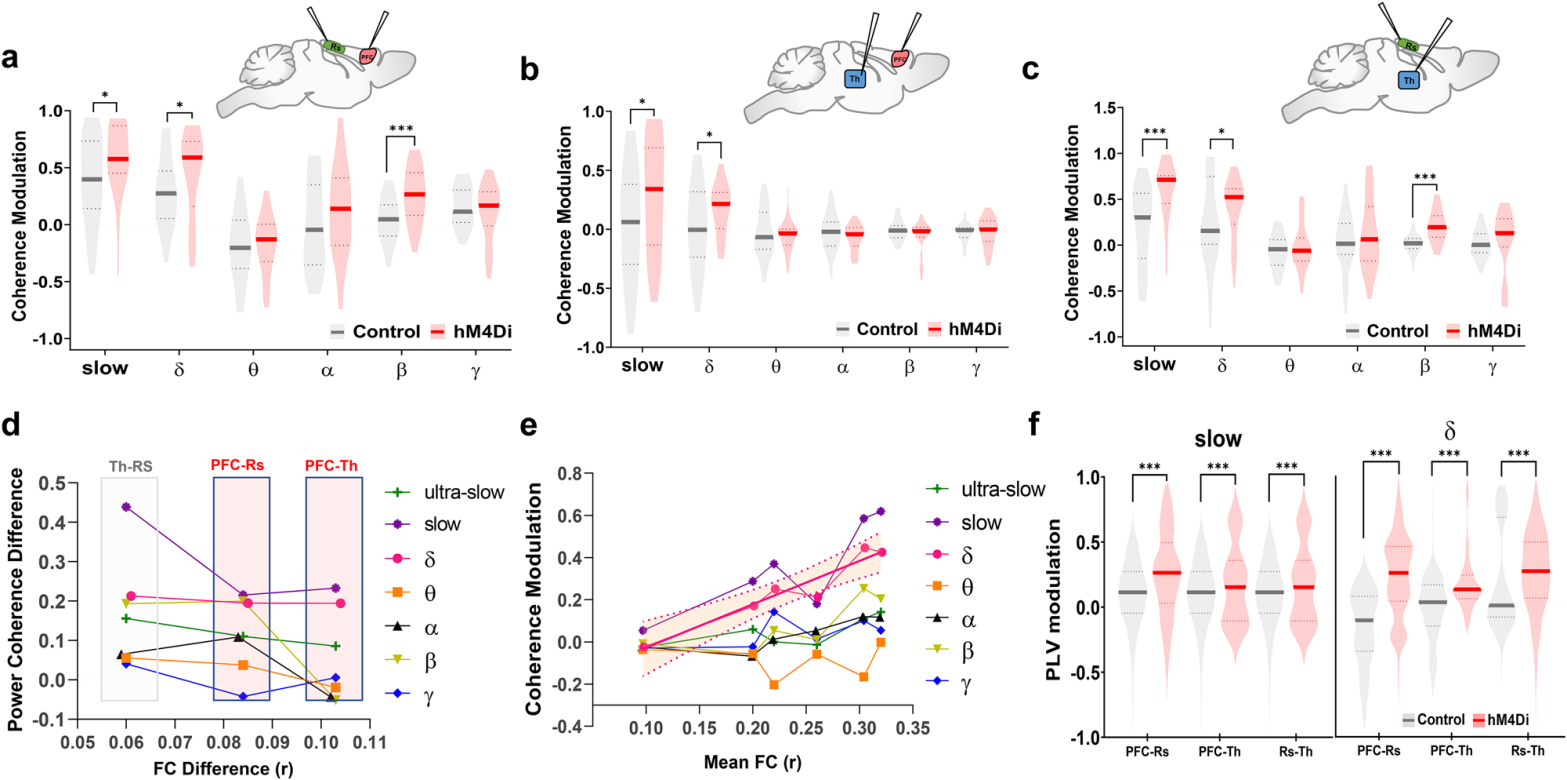
Chemogenetic inhibition of the PFC results in increased interareal slow oscillatory coherence. Baseline-normalized power coherence at different frequency bands for PFC-retrosplenial (**a**), PFC-thalamus (**b**), and retrosplenial-thalamus (**c**) electrode pairs (*q < 0.05, ***q < 0.001, one-sided Wilcoxon rank-sum test, FDR corrected for *n* = 50 and *n* = 40 statistically independent recordings from *n* = 5 hM4Di and *n* = 4 control mice, respectively). **d** Band-specific coherence and mean functional connectivity (FC) difference (hM4Di – Control) for all pairs of electrophysiologically-probed regions. Mean FC data were extracted for corresponding regional pairs (Fig. 3) during the CNO active time window in hM4Di and control animals. **e** Correlation between corresponding band-specific coherence and mean functional connectivity for all pairs of electrophysiologically-probed regions (PFC-Rs; PFC-Th; Rs-Th). Shaded area indicates 95% CI for δ. **f** Baseline-normalized phase coherence in slow and delta band between electrode pairs (Violin plots: thick lines represent median, dashed lines indicate 25th and 75th percentile, respectively; ***q < 0.001, two-sided Wilcoxon rank-sum tests, FDR corrected). PFC prefrontal cortex, PLV phase-locking value, Rs retrosplenial cortex, Th thalamus. Source data are provided as a Source Data file.

The increase in slow and δ band LFP power observed in the chemogenetically-inhibited PFC led us to hypothesize that the resulting rsfMRI overconnectivity could be driven by enhanced low-frequency neural coherence, as opposed to direct interareal communication via higher frequency neural oscillations^43–45^. To test this hypothesis, we carried out a new set of simultaneous multi-electrode LFP recordings in the PFC, retrosplenial, and centromedial (polymodal) thalamus of control and DREADD-transfected animals (Fig. 5). We selected these region pairs because they exhibit the highest functional overconnectivity in our chemo-fMRI study. Spectral-power quantifications after CNO administration revealed a reduction of γ and β LFP power in all the three recording sites, together with increased slow and δ LFP power in the PFC, but not in the retrosplenial cortex or centromedial thalamus of DREADD-transfected animals (Fig. S9a).

To explore the possible neural correlates of fMRI connectivity in terms of frequency-dependent neural communication, we next computed LFP power coherence between electrophysiological signals recorded at these electrode pairs and probed the presence of increased coupling between the recording sites. Notably, we found a clear CNO-induced increase in low-frequency LFP power coherence in PFC-Rs and PFC-polymodal thalamic areas in hM4Di-transfected animals (Fig. S9b). In keeping with rsfMRI evidence of increased thalamo-cortical connectivity upon PFC inhibition, enhanced low-frequency power coherence was also observed between the thalamus and retrosplenial cortex (Fig. S9b). Quantification of these changes in canonical LFP frequency bands revealed significantly increased power coherence in the slow and δ bands for all the three electrode pairs (Fig. 5a–c, *q* < 0.05, Wilcoxon rank-sum test, FDR corrected, all pairs). We next computed inter-areal coherence between the envelope of ϒ-band amplitude because ultra-slow (~0.1 Hz) variation in ϒ-band envelope has been linked to arteriole dynamics and suggested to be a contributing factor to rsfMRI coupling^41,46^. Mean coherence plots of ϒ-envelope over the ultra-slow frequency range (0.02–0.5 Hz^46^) revealed only marginally increased CNO-induced coherence in hM4Di expressing mice (Fig. S9c). In keeping with this observation, we did not find evidence of significantly increased ultra-slow ϒ-envelope coherence (integrated between 0.02 to 0.5 Hz) between any electrode pairs (q > 0.1, Wilcoxon rank-sum test, FDR corrected).

To link these oscillatory changes to the observed rsfMRI patterns, following the procedure described in Wang et al.^47^, we next compared the obtained electrophysiological coherence values with corresponding (separately-measured) interareal rsfMRI connectivity in hM4Di and control animals (Fig. 5d, e). We first compared between-group band-specific LFP coherence differences across all electrode pairs with corresponding interareal rsfMRI connectivity changes measured during the active CNO phase (Fig. 5d). These quantifications revealed that δ and slow LFP bands were the only frequency ranges exhibiting CNO-induced increased coherence between PFC-retrosplenial and PFC-thalamic electrode pairs, in agreement with the corresponding CNO-induced interareal rsfMRI connectivity increases (Fig. 5d). While considerably smaller, between-group ultra-slow LFP gamma-envelope coherence also revealed CNO-induced increases in all the three electrode pairs (Fig. 5d). Other LFP bands did not show full concordance with rsfMRI findings: for example, β band coherence increased after CNO administration in PFC-retrosplenial but not in PFC-thalamus electrode pairs. Similarly, no meaningful between-group changes in power coherence upon DREADD stimulation was observed in the θ and γ LFP bands (Fig. 5d). Further supporting a key contribution of slow and δ rhythms to the observed rsfMRI overconnectivity, we found that pairwise LFP coherence difference across all the recorded sites in these two bands exhibited a linear relationship with corresponding group-level pair-wise rsfMRI connectivity measured in both DREADD and control animals during the CNO active window (Fig. 5e, δ *R*^2^ = 0.92, *p* = 0.002; slow *R*^2^ = 0.73, *p* = 0.029, uncorrected). Only δ LFP coupling however retained a significant correlation with rsfMRI connectivity upon FDR correction (*q* = 0.008, FDR corrected). A much weaker correlation between band-specific LFP coherence and pairwise rsfMRI connectivity was apparent for all the other bands (Fig. 5e, *p* > 0.11 all bands). These findings were paralleled by the observation of similarly robust increased interareal δ- and slow-band LFP phase coherence, a widely used measure of functional synchronization in LFP/EEG studies^48^ (Fig. 5f). Taken together, these results corroborate a neural origin for our imaging findings and implicate increased slow and δ LFP coherence as a plausible neural driver of the observed rsfMRI over-synchronization.

**Fig. 6:**
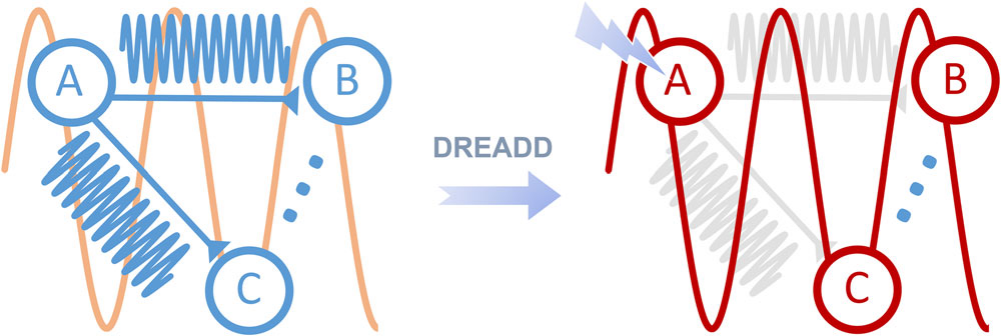
A schematic illustration of our findings. Chemogenetically inhibiting neural activity in cortical node A (i.e. PFC) reduces high-frequency direct interactions between the manipulated region and its targets (B and C), concomitantly producing higher entrainment of residual spiking activity with ongoing global low-frequency oscillations (node C). Under the assumption (supported by our data) that rsfMRI interareal connectivity is primarily driven by low-frequency neural synchronization, this interpretative framework predicts both the observed increase in interareal slow and δ LFP coherence, and the corresponding increase in interareal rsfMRI connectivity.

Our findings can be summarized in a simple illustrative scheme (Fig. 6) in which chemogenetic inhibition of the PFC (node A) reduces high-frequency local activity via a preferential suppression of spikes not locked to ongoing, global low-frequency rhythms. This in turns produces a breakdown of high-frequency communication between the manipulated area and its targets (B and C). At the same time, the residual firing activity in the inhibited node becomes more entrained with ongoing low-frequency fluctuations, here depicted as a global slow rhythm. The observed functional overconnectivity may then reflect the resulting increase in interareal slow and δ LFP coherence, which we have empirically found, under our experimental conditions, to predict the direction and magnitude of rsfMRI coupling better than the coherence of faster rhythms. Importantly, this model is also broadly consistent with examinations of LFP signatures of directionality of interactions between areas (Fig. S10). Specifically, using phase-lag quantifications as a measure of information flow directionality, we found that in the δ band PFC activity preceded in phase retrosplenial cortex activity, a finding consistent with previous reports of antero-posteriorly traveling δ wave activity in the anesthetized mouse brain^49^. Consistent with our hypothesis, after CNO administration the phase difference between PFC and retrosplenial cortex in DREADD-expressing animals was greatly reduced, implying that chemogenetic suppressions of local high frequency results in more synchronous and less phase-lagged δ activity. This finding suggests that after chemogenetic inhibition, PFC activity becomes more entrained with global, slow covarying rhythms.

## Discussion

Here we combine neural perturbations and mouse rsfMRI to investigate how rsfMRI topography reconfigures in response to targeted cortical inactivation. We report that chronic and acute neural inactivation of the mouse PFC can counterintuitively increase rsfMRI connectivity with DMN targets directly innervated by the inhibited area. Electrophysiological investigations revealed that chemogenetic inactivation of the PFC preferentially and robustly suppresses the firing activity that is not phased locked to highly excitable peaks of ongoing slow-oscillatory rhythms, leading to increased slow and δ band power, and enhanced interareal low-frequency coherence. These observations argue against a neural-independent origin for the observed hyper-connectivity, and implicate low-frequency neural rhythms in the establishment of the observed rsfMRI overconnectivity.

While critically shaped and constrained by underlying axonal connectivity^2–4^, spatiotemporal correlations in spontaneous rsfMRI activity can dynamically reconfigure in response to local perturbations. In-depth investigations of the reconfiguration patterns resulting from regional suppression of neural activity are of special interest. These allow for a targeted deconstruction of rsfMRI coupling and may offer opportunities to mechanistically interpret aberrant rsfMRI connectivity patterns in neurological conditions characterized by loss of cortical function^8,10,50^. Leveraging the recent implementations of chemo-fMRI in the mouse^20^, we causally probed how neural suppression of cortical activity affects brain-wide rsfMRI coupling. In contrast to theoretical^10^ and experimental^8^ evidence predicting that regional inactivation of a neural node would result in reduced functional synchronization with its direct anatomical targets, we found that both chronic and acute inhibition of the PFC can counterintuitively increase rsfMRI connectivity within thalamo-cortical substrates of the mouse DMN.

Our results advance our understanding of the principles underlying brain-wide rsfMRI coupling in two directions. First, we provide causal evidence that regional suppressions of brain activity does not necessarily lead to reduced neural and functional coupling between the inactivated area and its direct anatomical projection targets, but can result in increased rsfMRI connectivity via enhanced, less-localized slow oscillatory coherence. These observations point at a highly dynamic and non-monotonic relationship between structural and functional connectivity, underscoring a critical contribution of remote sources of large-scale neural covariation (e.g. δ and slow oscillations^40,51^) to the establishment of interareal rsfMRI coupling. This view is in keeping with correlational evidence of a dissociation between rsfMRI connectivity and underlying anatomical connections, such in the case of acallosal brains in which preserved bi-hemispheric connectivity has been repeatedly observed^7,14,15^. It should be emphasized here that these results should not be intended as a refutation of the structural foundations of rsfMRI connectivity, but rather the basis of an updated framework in which reciprocal interareal rsfMRI coupling can be strongly biased, or even overridden, by slow synchronized input from global rhythm generators.

Second, our results also provide an updated reference framework for the interpretation and reverse engineering of rsfMRI overconnectivity observed in brain pathology, especially in degenerative or neurological states characterized by rsfMRI overconnectivity. While a neural mass phenomenon like interareal rsfMRI coupling cannot be mechanistically dissected into discrete circuital elements, our observation of foci of overconnectivity in polymodal thalamic areas and the increased synchronization of these with larger cortical territories is intriguing, as it points at a putative involvement of higher order thalamic relay in the generation (or propagation) of the observed overconnectivity. This hypothesis would be consistent with the observation that full expression of δ and slow oscillatory activity requires thalamic participation^52,53^, and with recent evidence pointing at the presence of a prefrontal-thalamic loop involved in the generation and propagation of δ synchronization^11^. However, other modulatory mechanisms^12,54^ or subcortical substrates^55^ may similarly (or alternatively) play a role in the observed overconnectivity, and the identification of neural generators of rsfMRI coupling and their involvement in the reconfiguration of rsfMRI topography in response to local perturbations remain an open issue.

The observed increases in slow and δ band coherence upon inhibition of the PFC is in excellent agreement with previous reports of a robust association between spontaneous fMRI activity and slow oscillatory activity^40,47,51,56–59^. Our findings corroborate and expand these investigations by showing that reconfiguration of rsfMRI connectivity in response to local cortical inactivation is supported by increased interareal slow oscillatory coherence, a phenomenon reflecting DREADD-induced reduction of high-frequency firing, and concomitantly increased phase locking of residual spiking activity with ongoing global low-frequency oscillations. Recent work has linked ultra-slow (~0.1 Hz) variation in gamma-band amplitude to the vasomotor activity underlying intrinsic rsfMRI connectivity^41,46^, suggesting that this rhythm may serve as a possible primary generator of interareal rsfMRI coupling. Interestingly, while we found evidence of ultra-slow ϒ envelope coherence across functionally connected areas under basal conditions (Fig. S9c), the corresponding coherence increases upon chemogenetic manipulations were very small and did not correlate with corresponding pairwise rsfMRI overconnectivity. This finding suggests that, under the conditions of our manipulations, rsfMRI overconnectivity is most plausibly explained by increased canonical band (e.g. δ) low-frequency inter-areal coherence.

Although the light sedation protocol used in our measurements enhances slow oscillatory rhythms^60^, such a brain state is unlikely to be the primary reason for our observation of rsfMRI overconnectivity. Supporting this notion, increased rsfMRI connectivity between homotopic cortical regions has been observed in awake humans upon inhibitory transcranial magnetic stimulation (TMS) of the primary motor cortex^61,62^. Analgously increased rsfMRI overconnectivity and δ hyper-synchronization have also been observed patients with stroke and in early stage Alzheimer’s disease imaged in conscious conditions^19,63–65^. Given the highly dynamic nature of rsfMRI network activity, the sign and neurophysiological signatures identified in this study could however conceivably be affected by arousal levels. In this respect, a recent report of rsfMRI underconnectivity after chemogenetic inhibition of the rat postero-dorsal cingulate cortex is of interest in the light of a possible high-arousal state of these awake, restrained animals^66^. While the lack of electrophysiological recordings in this study prevents a direct comparison with our findings, high-arousal conditions are characterized by a robust shift towards high-frequency LFP activity and a possibly dominant contribution of direct feedforward interactions to interareal communication. Under these conditions, the synchronizing contribution of slow oscillatory activity may thus be largely reduced, and chemogenetic inhibition could thus produce qualitatively different effects. As robust methods for mapping rsfMRI activity in awake mice are becoming available^67^, systematic investigations of the effect of DREADD inhibition as a function of arousal state may help probe the plausibility of this model.

Interestingly, TMS investigations^61^ also show that excitatory pulse stimulations may produce rsfMRI de-synchronization, pointing at a possible general, inverse relationship between local cortical activity and interareal rsfMRI coupling. An analogous relationship might also be present in the mouse because, reconstituting the results of our control experiments, decreased rsfMRI connectivity has been recently observed with excitatory DREADD stimulation of somato-motor areas in lightly anesthetized animals^68^. This relationship however requires further corroboration, as KORD-based chemogenetic inhibition of the cingulate cortex was instead reported by others to produce rsfMRI underconnectivity in anesthetized mice^69^. These inconsistencies reveal the critical need to couple perturbational rsfMRI studies with electrophysiological recordings to confirm the effect of the designated manipulation, and relate changes in rsfMRI connectivity to underlying neural rhythms.

A strength of our approach is the use of translationally-relevant fMRI-based readouts enabling a possible extrapolation of experimental findings to analogous human research. However, some limitations need to be recognized when assessing the generalizability of our results across species and conditions. First, the magnitude and direction of rsfMRI connectivity produced by neuronal inhibition could be manipulation specific^27^, an effect that in the case of chemogenetics might also be compounded by possible off-target effects of commonly used chemogenetic actuators^70^. By employing unlike others^8,66,69^, a design in which CNO is administered under all conditions to all experimental groups, we were able to rigorously control any confounding effect of this actuator via a direct contrast of CNO-treated control and DREADD-expressing animals. As a result, the effects we report in the manuscript are to be attributed to the sole DREADD activation. The observation that chronic inhibition with Kir2.1 reconstitutes the overconnectivity obtained under DREADD manipulation increases our confidence in the validity of our mechanistic inferences. Second, it should be emphasized that the mechanisms that we report here may not be necessarily applicable to awake, behaving conditions, as task-dependent cognitive and sensory processing may strongly and dynamically bias brain rhythms and interregional coupling beyond what could be inferred in the resting, sedated brain. This aspect however does not diminish the translational impact of our findings, owing to the prominent use of resting-state fMRI to map functional network activity and our current inability to back-translate rsfMRI signatures of brain dysfunction into physiologically interpretable events.

In conclusion, the present work provides causal evidence that inhibition of a cortical region can lead to counterintuitively increased patterns of rsfMRI connectivity, an effect mediated by increased interareal slow-frequency coherence. These findings point at a non-monotonic relationship between regional cortical activity and network-wide rsfMRI connectivity, and define testable network-level mechanisms for the emergence of rsfMRI overconnectivity in clinical conditions characterized by loss of cortical function.

## Methods

### Ethical statement

All in vivo experiments were conducted in accordance with the Italian law (DL 26/214, EU 63/2010, Ministero della Sanità, Roma). Animal research protocols were reviewed and consented by the animal care committee of the University of Trento and Italian Ministry of Health (authorization no. 852/17 to A.G.). All surgical procedures were performed under anesthesia.

### Animals

Kir 2.1, hSyn-hM4Di inhibition and CamkII-hM3Dq stimulation studies were carried out in adult (6 week old) male C57Bl6/J mice (Jackson laboratories, Stock No: 000664). hM4Di inhibition of parvalbumin-positive neurons was carried out in adult (6 week old) transgenic animals expressing Cre recombinase in parvalbumin-positive GABAergic neurons (B6.129P2-Pvalb^tm1(cre)Arbr^/J, Jackson laboratories, Stock No 017320)^71^. Mice were group housed in a 12:12 h light-dark cycle in individually ventilated cages with access to food and water ad libitum and with temperature maintained at 21 ± 1 °C and humidity at 60 ± 10%.

### Anatomical definition of mouse medial prefrontal cortex

Our anatomical definition of mouse medial prefrontal cortex (PFC) reflects recent neuroanatomical^72^ and cytoarchitectural cross-species comparisons^73^, according to which the mouse PFC comprises a prelimbic region, corresponding to primate Brodmann area 32 (A32), the anterior cingulate cortex, corresponding to Brodmann area A24b, and the infralimbic cortex, corresponding to Brodmann area A24a. Our viral manipulations were therefore aimed to inhibit an anatomical ensemble comprising all the abovementioned regions at the following coordinates, expressed in millimeter from Bregma: 1.7 from anterior to posterior, ±0.3 lateral, −1.7 deep^74^.

### Viral injections

Mice were anesthetized with isoflurane and head-fixed in a stereotaxic apparatus (Stoelting). Injections were performed with a Hamilton syringe mounted on Nanoliter Syringe Pump with controller (KD Scientific), at a speed of 0.05  μl/min, followed by a 5–10 min waiting period, to avoid backflow of viral solution. To prevent layer- or cell-type-specific expression^28^, all in vivo viral transductions were carried out using high-titer (>10^13^ vg/mL) viral suspensions. The following injections volumes were employed: 300 nL (AAV8-hSyn-hM4D(Gi)-mCherry and AAV8-hSyn-GFP; http://www.addgene.org) or 2 μL (AAV8-hSyn-MYC-mKir2.1(E224G/Y242F)-IRES-GFP, Xue et al.^23^, http://www.vectorbiolabs.com or AAV8-hSyn-GFP http://www.addgene.org), or 1 μL (AAV8-CamkII-hM3D(Gq)-mCherry; http://www.addgene.org) or 300 nL (AAV9-hSyn-DIO-hM4D(Gi)-mCherry; http://www.addgene.org) of viral suspension were injected bilaterally in the mouse PFC (see coordinates above). Control groups for both CamkII-hM3D(Gq) and PV-hM4D(Gi) were obtained by sham-injecting genotype-matched littermates. rsfMRI or electrophysiological recordings were carried out no sooner than three weeks after the injection to allow for maximal viral expression.

#### rsfMRI acquisitions

The animal preparation protocol was recently described in great detail^33,75,76^. Briefly, mice were anesthetized with isoflurane (5%, induction), intubated, and artificially ventilated (2%, surgery). The left femoral artery was cannulated for continuous blood pressure monitoring. At the end of surgery, isoflurane was discontinued and substituted with a shallow halothane regimen (0.75%) to obtain light sedation and to preserve cerebral blood flow auto-regulation^77^. Ventilation parameters were adjusted to maintain normo-physiological p_a_CO_2_ (<40 mmHg) and p_a_O_2_ levels (>90 mmHg, corresponding to >98% hemoglobin saturation). To probe the generalizability of our findings, we also repeated our inhibitory chemo-fMRI manipulations under a combination of medetomidine and low-dose isoflurane (0.05 mg/kg bolus and 0.1 mg/kg/h IV infusion, plus 0.5% isoflurane^35,36^).

rsfMRI data acquisition commenced 30 min after isoflurane cessation. Functional images were acquired with a 7 T MRI scanner (Bruker, Ettlingen) equipped with a BGA-9 gradient set (380 mT/m, max. linear slew rate 3,420 T/m/s), using a 72 mm birdcage transmit coil and a 4-channel solenoid coil for signal reception^78^. The scanner was operated using Paravision 6.01 software (Bruker, Ettlingen). Single-shot BOLD rsfMRI time series were acquired using an EPI sequence with the following parameters: TR/TE 1000/15 ms, flip angle 60°, matrix 98 × 98, FOV 2.3 × 2.3 cm, 18 coronal slices, slice thickness 550 µm, bandwidth 250 KHz. rsfMRI acquisition with Kir2.1-transduced (AAV8-hSyn-MYC-mKir2.1(E224G/Y242F)-IRES-GFP, *n* = 16) and control mice (AAV8-hSyn-GFP, *n* = 19) encompassed 35-min-long timeseries, corresponding to 2100 volumes.

Chemo-fMRI acquisitions comprised two consecutive rsfMRI timeseries, encompassing 1800 volumes (30 min) and 2100 volumes (35 min), respectively. CNO (2 mg/kg, Sigma Aldrich) was injected intravenously fifteen minutes (volume #900) after the start of the first scan. The first 900 fMRI volumes of this first timeseries scan were used as pre-CNO baseline rsfMRI reference in time-resolved analyses. Based on the pharmacokinetic profile of CNO, the post CNO window was split into temporal domains as follows: the first 15 min post injection (900 volumes) were considered part of a drug equilibration window, while the following 35 min (2100 volumes) were considered to cover the DREADD active time window^30^. All group comparisons in the chemo-fMRI study were carried out within this latter time window, unless otherwise stated. After post-mortem analyses of viral expressions, a total of *n* = 15 hM4Di and *n* = 19 GFP-transduced animals were retained for analyses.

#### Image preprocessing and analysis

Raw rsfMRI timeseries were preprocessed as follows. The initial 120 volumes of the time series were removed to allow for thermal gradient equilibration. Data were then despiked, motion corrected, and spatially registered to a common reference template^33,75^. Motion traces of head realignment parameters (3 translations + 3 rotations) and mean ventricular signal (corresponding to the averaged BOLD signal within a reference ventricular mask) were used as nuisance covariates and regressed out from each time course. All rsfMRI time series also underwent band‐pass filtering within a frequency window of 0.01–0.1 Hz (halothane) or 0.01–0.25 Hz MED-ISO^35^, followed by spatial smoothing with a full width at half maximum of 0.6 mm. To control for the effects of global fMRI signal regression on the mapped changes, all rsfMRI timeseries were also recomputed by regressing average fMRI signal within an intracerebral mask.

rsfMRI connectivity of the mouse DMN in Kir2.1 and chemo-fMRI scans was probed using a seed-based approach. In the case of the chemo-fMRI study, this quantification was carried out during the CNO active time window. A 5x5x2 seed region was selected to cover the PFC areas targeted by viral injections. Voxel‐wise intergroup differences in seed-based mapping were assessed using a 2‐tailed Student’s *t* test (|t| > 2, *p* < 0.05) and family‐wise error (FWE) cluster‐corrected using a cluster threshold of *p* = 0.050 as implemented in FSL (https://fsl.fmrib.ox.ac.uk/fsl/). The antero‐posterior connectivity profile of the DMN was assessed by computing Person correlation between the PFC seed abovementioned and a series of 6 × 6 × 2 voxel seeds placed along the midline extension of the cingulate and retrosplenial cortices^26^. Quantification of cortico-thalamic connectivity was carried out with respect to a meta-regional parcellation of the mouse cortex in volumes-of-interest. To rule out a possible confounding contribution of spurious neurovascular changes in CNO-induced rsfMRI connectivity alterations, we calculated and statistically compared the characteristic hemodynamic response function^26,79^ between Kir2.1 and control mice, and between hM4Di-expressing and control mice upon CNO-administration (active phase).

Whole-brain connectivity in hM4Di and Control mice was calculated across a set of volumes of interest recapitulating anatomical areas of the Allen brain atlas. The anatomical probed area were selected according to their coverage of previously characterized network systems of the mouse brain^21,75,78^: TH: thalamus (thalamus polymodal association cortex related, Thalamus Sensory-Motor cortex related); STR: striatum (striatum dorsal region left, striatum dorsal region right, striatum ventral region left, striatum ventral region right); LCN: lateral cortical network (LCN: primary motor cortex left, primary motor cortex right, primary somatosensory cortex left, primary somatosensory cortex right, secondary somatosensory cortex left, secondary somatosensory cortex right, Lateral septal complex left, lateral septal complex right); HCP: hippocampus (Ammon’s horn left; Ammon’s horn right, dentate gyrus left; dentate gyrus right, Entorhinal area left, entorhinal area right, subiculum left, subiculum right); DMN: default mode network (anterior cingulate area; Infralimbic area, secondary motor cortex left, secondary motor cortex right, orbital area, prelimbic area, posterior parietal association areas left, Posterior parietal association areas right, retrosplenial area);

To relate the strength of underlying anatomical connectivity to the regions exhibiting increased rsfMRI connectivity with voxel resolution, we extracted outgoing projections from the affected PFC regions using a spatially-resampled (0.027 mm^3^) version of a voxel scale model of the Allen Brain Institute structural connectome^4^. We then plotted the strength of PFC-departing structural projections against the corresponding between-group difference in rsfMRI connectivity using the cluster-corrected difference map and assessed differences in the distribution of overconnected areas with respect to all the brain voxels using a Wilcoxon rank-sum test.

To quantify the contribution of distinct thalamic subregions to overall group differences, we used k-means clustering to partition voxels within the thalamus, based on whole-brain rsfMRI group-difference obtained using the PFC seed as recently described^80,81^. This approach revealed two major thalamic clusters, one medial and one bilateral partition encompassing sensory areas. Seed-based functional connectivity was subsequently computed for each of the two-resultant k-means clusters independently, and the resulting functional connectivity maps were compared and quantified across cortical VOIs.

### Electrophysiological recordings

Electrophysiological recordings were carried out in animals subjected to the same animal preparation and sedation regime employed for rsfMRI mapping^14,77^. Briefly, mice were anesthetized with isoflurane (5% induction), intubated, artificially ventilated (2% maintenance), and head-fixed in a stereotaxic apparatus (Stoelting). The tail vein was cannulated for CNO injection. To ensure maximal consistency between viral injections and recording site, the skull surface was exposed and an insertion hole in the right PFC was gently drilled through the skull corresponding to the location of prior viral injection point. A single shank electrode (Neuronexus, USA, interelectrode spacing 1–2.5 mm) was next inserted through the overlying dura mater by a microdrive array system (Kopf Instruments, Germany) at an insertion rate of 1 µm/min to reach the same stereotaxic coordinates employed for viral injection. The site receptive fields were plotted manually and the position and size of each field were stored together with the acquisition data. After electrode insertion, isoflurane was discontinued and replaced by halothane at a maintenance level of 0.75% to induce rsfMRI-comparable sedation. Electrophysiological data acquisition commenced 1 h after isoflurane cessation. Such transition time was required to ensure complete washout of isoflurane anesthesia and avoid residual burst-suppressing activity associated with extended exposure to deep anesthetic levels.

Neural activity was next recorded in consecutive 5 min time bins to cover a 15 min pre-injection time window, and a 60 min post CNO timeframe in *n* = 5 hM4Di and *n* = 5 GFP-expressing mice. Signals were amplified using an RHD 2000 amplifier system (Intan Technologies, RHD Recording Controller Software, v2.09) to acquire electrophysiological data at a sampling rate of 20 kHz. For CamkII-hM3D(Gq) (*n* = 4) and PV-hM4D(Gi) (*n* = 6) experiments we acquired respectively *n* = 4 and *n* = 7 control animals. In the case of control Kir2.1 recordings, a four shank electrode was inserted along the coronal plane to bi-hemispherically cover the right (Kir2.1-expressing) and left (GFP-expressing) PFC (*n* = 4). The left region served as internal reference control to better assess the efficacy of Kir2.1 neural inhibition. Electrophysiological signals were then recorded into 5 min time bins to cover a 35-min time-window.

To measure multi-electrode coherence, three electrodes were inserted in key cortical and subcortical substrates identified as overconnected in our chemo-fMRI mapping in *n* = 4 hM4Di and *n* = 5 GFP-expressing mice. A multi-probe micromanipulator (New-Scale Technologies) was used to insert three 16 channels single shank electrode (Neuronexus, USA, interelectrode spacing 1–2.5 mm) in the right prefrontal cortex, centromedial thalamus, and retrosplenial cortex, respectively. Representative electrode locations are reported in Fig. S11, corresponding to the following stereotaxic coordinates: PFC 1.7 mm AP, ±0.3 mm ML, −1.7 mm DV; mediodorsal thalamus: −1.7 mm AP, −0.3 mm ML, −3.5 mm DV (5° insertion angle); retrosplenial cortex: −2.4 mm AP, −0.3 mm ML, −1.3 mm DV. To reduce tissue damage, an insertion rate < 1 µm/min was employed, allowing for a 30-min equilibration every 400 µm traveled. Ground electrodes were put in contact with the cerebral brain fluid through a window drilled in the skull. Signals were then recorded into 1 min time bins to cover a 15-min pre-injection baseline and a 40-min post CNO time window.

### LFP and multi-unit activity

To compute the LFP signal, raw extracellular recordings were first down-sampled to 4 kHz, then band-pass filtered to 1–250 Hz using a two-step procedure^82^. Briefly, raw timeseries were first low-pass filtered using a 4th order Butterworth filter with a cut-off frequency of 1 kHz. The resulting timeseries were next down-sampled to 2 kHz, then again filtered using a Kaiser window filter between 1 Hz to 250 Hz (with a sharp transition bandwidth of 1 Hz, passband ripple of 0.01 dB and a stop band attenuation of 60 dB) and then resampled at 1 kHz. All the filtering was applied both in forward and backward temporal direction to avoid any phase transitions due to filtering.

To compute multi-unity activity (MUA) we again followed the procedure described in ref. ^82^. Briefly, we computed a band-passed high-frequency signal using a 4th order Butterworth filter with a <100 Hz cut off frequency, then band-pass filtered between 400 and 3000 Hz using a Kaiser window filter (with transition band of 50 Hz, stopband attenuation of 60 dB, and passband ripple of 0.01 dB). From the high-frequency signal we detected spike times using a spike detection threshold corresponding to 4-times the median of the high-frequency signal, then divided by 0.6745 as suggested in Quiroga et al. (2004). Spikes were considered to be biologically plausible, and as such retained in these computations, only if occurring more than 1 ms apart.

To quantify effectiveness of Kir2*.1* in suppressing spontaneous activity, firing rate was computed (in units of spikes/s) by dividing the number of spikes per electrode by the recording duration in seconds. The resulting spike rates were averaged across the channels corresponding to the virally targeted or control region. The average spike rate for four subjects was next tested against each other using paired *t* test. To assess the effect of chemo-fMRI manipulations, spiking activity was computed in experimental and control animals as described above and segmented into one-minute bins. We next computed the channel-averaged firing rate for each segment, and normalized this value with respect to the firing rate recorded during baseline (pre-CNO) period. The resulting baseline-normalized firing rate index was then used to assess changes in spiking rate upon CNO injection in the two experimental cohorts.

To determine the time lag at which different firing rate samples from the same channels could be considered as approximately statistically independent, we computed for each subject the autocorrelation of the channel-averaged firing rate for each subject and computed the time lag after which the autocorrelation function drops below the 95th percentile. We then, for each subject, retained samples of the baseline-normalized firing rate at different times separated by the above obtained lags. We next separately analyzed the baseline-normalized rates in three different periods: 0–15 min (transient time), 15–35 min, and 35–55 min after CNO the injection (active time). We pooled all the retained data points in these windows both over time and over subjects and then compared the median between the two populations using a two-sided Wilcoxon rank-sum test. The obtained *p*-values were corrected using a Benjamini–Hochberg FDR correction.

LFP spectrograms were computed using a Fourier transform with a Kaiser window with a frequency resolution of 0.15 Hz, temporal resolution of 6 s, and with 50% overlapping windows. Spectrograms and their differences were smoothed in time with the resolution of 30 s, and in frequency with the resolution of 1 Hz using a median filter. To quantify the effect of CNO on LFP rhythms, we computed a spectrogram modulation index as follows. First, we computed the channel-averaged spectrograms for the duration of the baseline recording. Next, we averaged time-frequency spectral profiles over time, resulting in frequency-resolved spectral profiles. The effect of CNO was next assessed by computing a modulation index, defined as the ratio of channel-averaged spectrogram after injection minus baseline, time-averaged spectrogram, and the sum of the same quantities, for every time window and for every frequency. This modulation index ranges between -1 and 1 and describes the changes due to drug injection over time for each frequency.

To obtain a statistical assessment of CNO effects across groups and bands, we computed the autocorrelation of spectrograms for every subject at every frequency, using (as for our assessment of firing rate) only spectrograms computed at time points far enough to be approximately statistically independent. The time lag to determine such approximately independent points was identified as the interval after which the spectrum’s autocorrelation function dropped below the 95th percentile. We next computed the median of the modulation index over different frequency bands defined as follows: slow (0.1–1 Hz), δ (1–4 Hz), θ (4–8 Hz), α (8–12 Hz), β (12–30 Hz), and ƴ (30–70 Hz). Data within each band were pooled over uncorrelated time points (determined as above by taking time samples separated by lags at which autocorrelation became negligible) and over subjects, and the population medians were compared using two-sided Wilcoxon rank-sum tests, followed by FDR correction.

#### Multielectrode coherence

Channel-averaged spectrograms were preprocessed as described above. Raw spectral power coherence was assessed by computing the magnitude of squared coherency using Welch’s overlapped averaged periodogram method^83^ with a 50% overlapping window of 2 s length. Coherence was calculated for every 60-s bin of channel-averaged recordings. Temporal smoothing was carried out using a 60-s median filter as described above. CNO spectrograms (30–40 post CNO injection window) were normalized with respect to the last 3 min of pre-CNO baseline using a modulation index as before. To obtain a quantifiable assessment of the CNO effect across different groups and bands, data within each band were pooled into 60-s bins. Subject and the population medians were compared for each region separately using one-sided Wilcoxon rank-sum tests, followed by FDR correction. The use of 60 s bins was motivated by estimations of the time lags at which autocorrelation of electrophysiological signals drops below the 95th percentile value (and thus samples are approximately independent) as described above.

To assess changes in ultra-slow fluctuations in the ϒ band envelope of LFP, following the work from Nir et al.^41^, we computed LFP data spectrogram for the last 10 min of baseline and 30–40 min of post injection window, using a moving window of 2 s width and 0.75 percent overlap, resulting in an estimation of the resulting ultra-slow power for each 0.5 s interval. The ϒ-band envelope was computed as the integrated power of the spectrograms in the ϒ band 30–70 Hz^46^. The power spectrum of this time series was next computed, after correction for 1/f component^46^, revealing a peak in the 0.02–0.05 Hz region. Interareal coherence was then computed as described above for both experimental and control mice. To assess the effect of chemogenetic manipulations, we defined a coherence modulation index as described above for other frequency bands. Between group statistical assessments were carried out using two‐sided Wilcoxon rank‐sum tests, followed by FDR correction.

To quantify interregional phase coupling, LFP data were first filtered using a third order Butterworth filter in delta band, and the instantaneous phase of each channel was computed by taking the phase of the analytical signal resulted from the Hilbert transform. For all possible pairs of channels belonging to two different regions, we next computed the corresponding Phase Locking Value (PLV) as follows:

$${{{{{\rm{PLV}}}}}}=\frac{1}{{{{{{\rm{N}}}}}}}\left|\mathop{\sum }\limits_{j=1}^{N}{e}^{i\left({\theta }_{{ch}1}\left({t}_{j}\right)\,-\,{\theta }_{{ch}2}\left({t}_{j}\right)\right)}\right|\,$$

where N is the number of data points in time and ${\theta }_{{ch}1}\left({t}_{j}\right),{\theta }_{{ch}2}\left({t}_{j}\right)$ are the instantaneous phase of the LFP of channel 1 and 2 at time j. The PLV value for each pair during the 30–40 min post CNO was next normalized with respect to the PLV value of the last 3 min of the pre-CNO baseline using a modulation index and was pooled over channel pairs and animals. The obtained population medians of the control and experimental group for each region pair were next compared using two-sided Wilcoxon rank-sum tests, followed by FDR correction.

#### Power spectrum of simulated spiking activity

To understand whether the increase in δ and slow LFP power observed with DREADD-induced inhibition could be explained by selective elimination of spikes not occurring at the preferred, most excitable, phase of the considered oscillation, we simulated 10 s epochs of spikes locked to a cosine “LFP” wave oscillating at 1 Hz. In the first scenario, representing the DREADD conditions, we generated 100 spikes (rate of 10 spikes per second) tightly distributed around the preferred phase π with values taken from a von Mises distribution with an extremely high value (20) of the concentration parameter. For the second case (representing sham condition), we added 100 more spikes distributed almost randomly across phases, with values taken from a von Mises distribution with an extremely low value (0.5) of the concentration parameter. We then smoothed spike temporal components with a Gaussian kernel (100 ms width) and computed the power spectra of the resulting spike train with the multitaper method (Matlab pspectrum function).

#### Relationships between spikes and LFP phases

We computed at each time point the phase of the LFP in a given frequency band as the angle of the Hilbert transform of the band-passed LFP. We considered for this analysis the slow and δ bands. Signal in each band was band-passed using a 3rd order Butterworth filter. To measure the locking of spikes to specific phases of each band, we considered the distribution of the LFP phase values at the time of each MUA spike. From this distribution, we computed the phase locking value (PLV) as 1 minus the circular variance of this distribution (Methods Section “Multielectrode coherence”). PLV ranges from 0 (no locking or relationship between phase and spike times) to 1 (perfect locking of spikes to a certain phase). As for other quantities, we also computed a PLV modulation index as the difference of PLV values between the active-drug and baseline periods divided by the sum of PLV values in the active-drug and baseline periods. A two-sided Wilcoxon was used to check if the median PLV modulation index is higher for the experimental data with respect to control data.

Violin plot representations of all electrophysiological analyses include individual statistically independent points (see above) used for intergroup comparisons. Plots were generated using Prism Graphpad 9.1. Plot truncation at extremities is introduced by the software to avoid representation of kernel density-related fictitious values above the highest data value or below the smallest.

#### LFP-LFP phase differences

To characterize the phase differences across areas, we computed the distribution across time of the difference in the instantaneous phase (computed as explained above) of PFC and Rs electrode pairs. From this distribution, we considered a channel pair to have significant PLV (see “Multielectrode coherence” section above) if its PLV value exceeded the 95th percentile of surrogate PLV distribution obtained by shuffling LFPs in time prior to filtering, and if the same PLV value was also greater than 0.1. We report in Fig. S10 the distribution of preferred phase differences (the circular mean of the phase difference distribution) for these significant channels. In the same figure, we also report (as thick lines) the values of the circular mean across all significant channels in each condition. We here report only the results of retrosplenial and PFC electrode pairs in the δ band, because this was the only combination exhibiting very stable mean circular phases across all baseline conditions and during the active time of the control injection. Circular and statistics were all computed with CircStat package^84^.

### Reporting summary

Further information on research design is available in the Nature Research Reporting Summary linked to this article.

## Supplementary information

Supplementary Information

Reporting Summary

## Data Availability

The rsfMRI and electrophysiology data generated in this study are under active use by the reporting laboratory; all data presented in this manuscript are available by reasonable request. Source data are provided with this paper. BOLD fMRI parametric maps are available for download at: https://data.mendeley.com/datasets/2b3n86gr25/1.

## Source data

Source Data

## Code availability

The code used for preprocessing and analyzing mouse rsfMRI data is available at https://github.com/functional-neuroimaging/rsfMRI-preprocessing and https://github.com/functional-neuroimaging/rsfMRI-global-local-connectivity. Code for analyses of epelctrophysiological recordings is available at https://github.com/functional-neuroimaging/PFC-chemo-inhib.
