# Supplementary material for: Increased fMRI connectivity upon chemogenetic inhibition of the mouse prefrontal cortex
Source: Nat Commun. 2022 Feb 25;13:1056. doi: 10.1038/s41467-022-28591-3 (PMC8881459; doi:10.1038/s41467-022-28591-3)
Supplement: Supplementary file 1 — Supplementary Information [file 41467_2022_28591_MOESM1_ESM.pdf]

Supplementary material

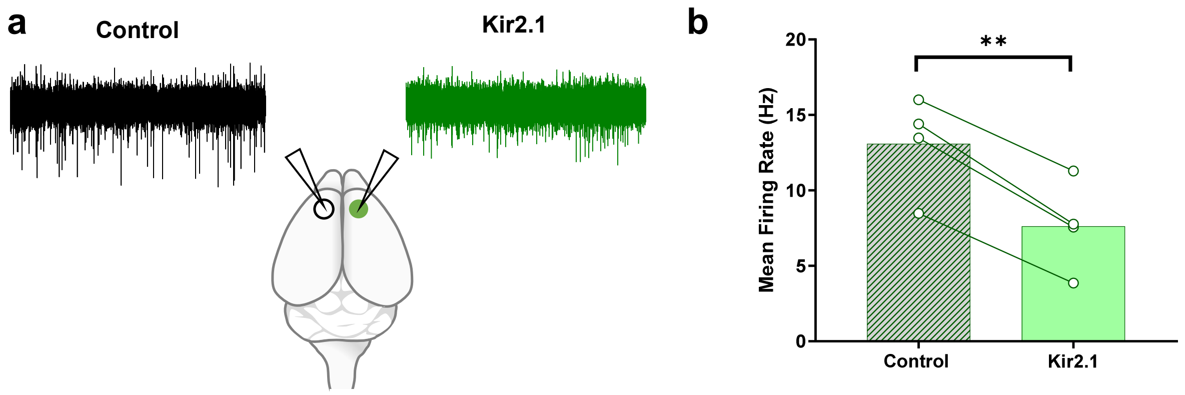

Figure S1. Overexpression of the potassium channel Kir2.1 in the PFC reduces spontaneous neural activity. (a) Experimental design: Kir2.1 injection was performed unilaterally in the right PFC. A viral vector encoding GFP was injected in the contralateral area. Electrophysiological recordings were carried out bilaterally using a four-shank electrode. (b) Mean spontaneous firing rate for the control side (no Kir2.1 expression), and the side expressing Kir2.1. (n=4; ** p = 0.002, two-sided paired t-test). Source data are provided as a Source Data file.

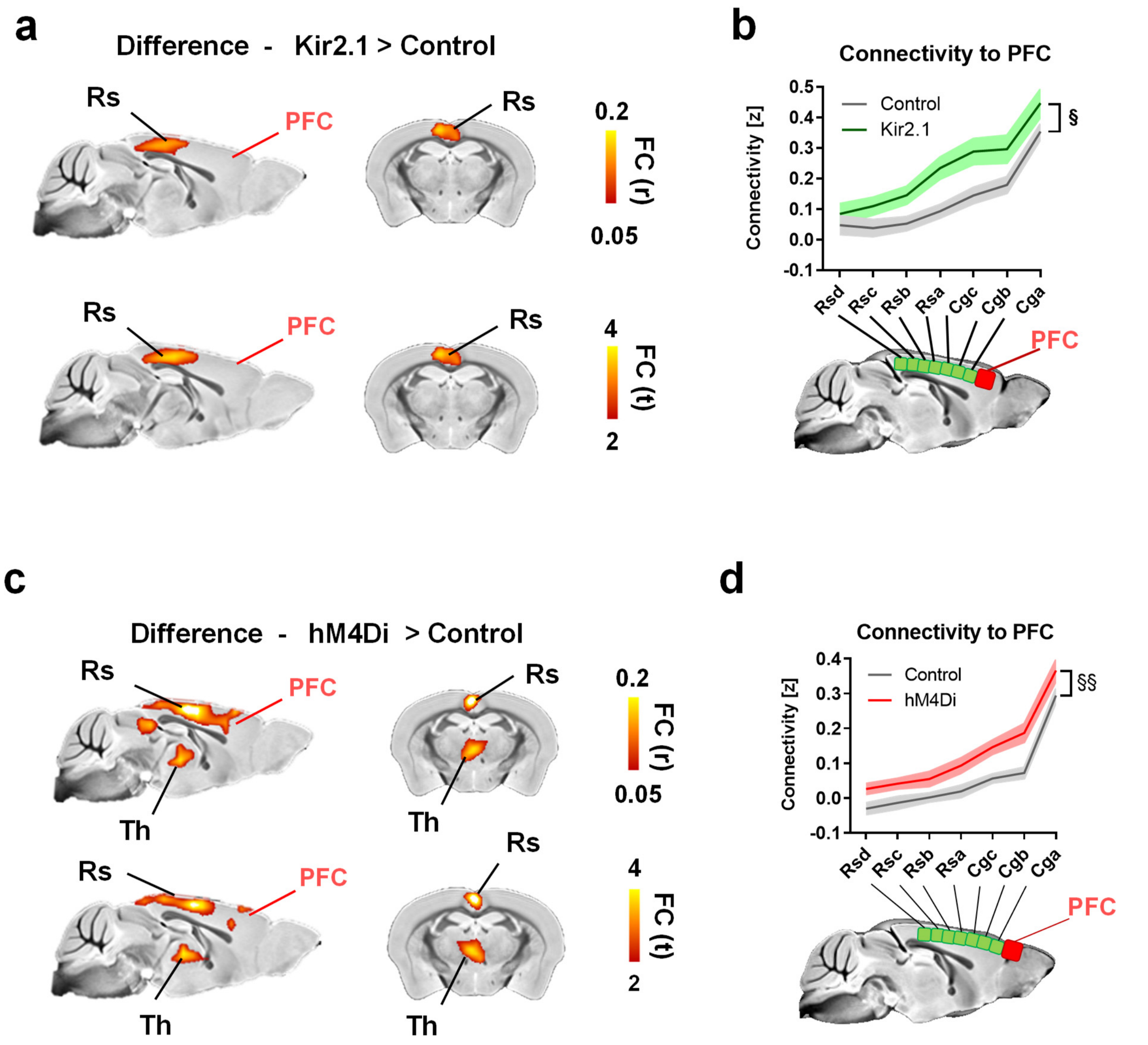

Figure S2. rsfMRI overconnectivity in Kir2.1 and DREADD-expressing mice persists after global fMRI signal regression. (a) Between-group difference maps (Pearson's r, and corresponding T stat difference maps). rsfMRI connectivity was here computed after fMRI global signal regressions. (b) Antero-posterior profiling of rsfMRI connectivity of the PFC along the midline axis of the mouse DMN in the two cohorts revealing consistent overconnectivity in Kir2.1 mice (§ $p = 0.013$, 2-way ANOVA repeated measurements, genotype effect, $n = 16$ and $n = 19$ Kir2.1 or GFP-expressing mice, respectively). (c-d) rsfMRI overconnectivity in hM4Di mice is not affected by rsfMRI global signal regression (c) Between-group difference maps (Pearson's r, and corresponding T stat difference maps, two-sided). (d) Antero-posterior profiling of rsfMRI connectivity of the PFC along the midline axis of the mouse DMN in the two cohorts upon fMRI global signal regressions (§§ $p < 0.001$, 2-way ANOVA repeated measurements, genotype effect, $n = 15$ and $n = 19$ hM4Di or GFP-expressing mice, respectively). Data in (b) and (d) are presented as mean values ± SEM. Cg: cingulate cortex; PFC: prefrontal cortex, RS: retrosplenial cortex, TH: Thalamus; FC: functional connectivity. Source data are provided as a Source Data file.

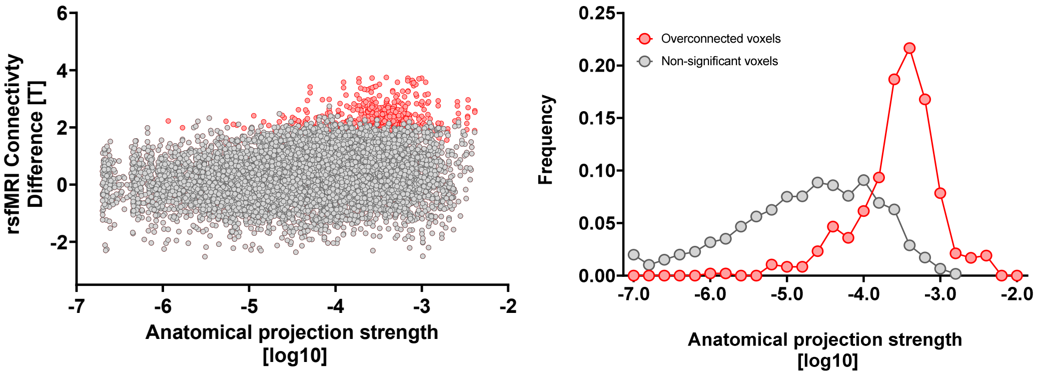

Figure S3. Voxels exhibiting rsfMRI overconnectivity upon chemogenetic inhibition of the PFC are robustly innervated by the PFC. Left: scatter plot illustrating intergroup differences in rsfMRI connectivity as a function of PFC structural connectivity strength. Note that all significantly overconnected voxels (red) contain robust axonal projections from the PFC. Right: distribution of voxels exhibiting the most significant rsfMRI connectivity (red) and those that are not affected (grey). Source data are provided as a Source Data file.

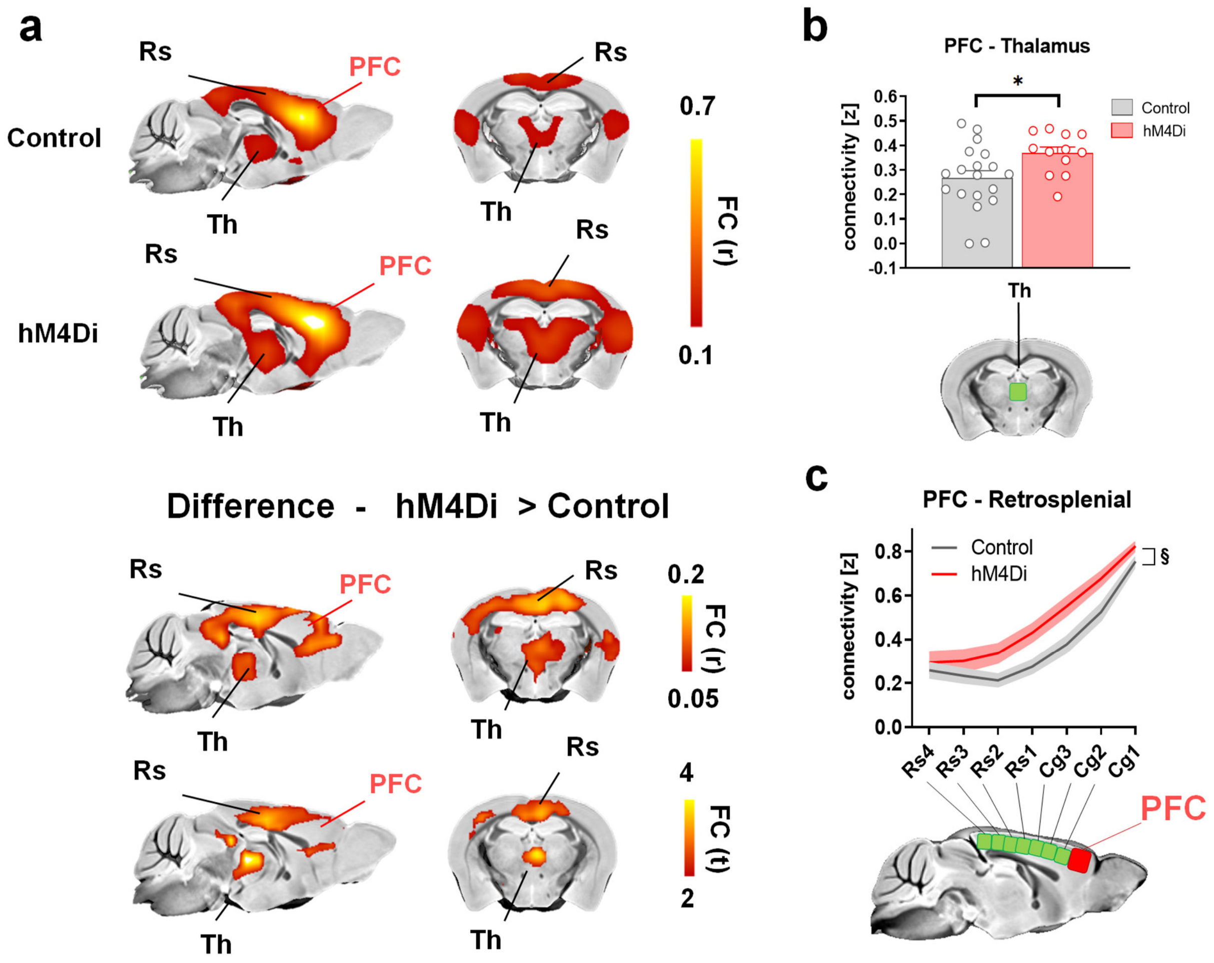

Figure S4. Chemogenetic inhibition of the mouse PFC under medetomidine-isoflurane anesthesia recapitulates the rsfMRI overconnectivity profile observed in halothane-anesthetized mice. (a) Seed-based connectivity of the PFC in control (hSyn-GFP n = 19) and experimental (hSyn-hM4Di, n = 12, top) animals (top) and corresponding between-group connectivity difference map (Pearson's r, and T stat, respectively, bottom). (b) Thalamo-cortical rsfMRI overconnectivity in hM4Di expressing mice (*p = 0.03, two-sided t test, n = 12 and n = 19 hM4Di or GFP-expressing mice, respectively). (c) Antero-posterior profiling of rsfMRI connectivity of the PFC along the midline axis of the DMN in the two cohorts (§ p = 0.012, 2-way ANOVA repeated measurements, genotype effect, n = 12 and n = 19 hM4Di or GFP-expressing mice, respectively). Data in (b) and (c) are presented as mean values ± SEM. Cg: cingulate cortex; PFC: prefrontal cortex, RS: retrosplenial cortex; Th: Thalamus; FC: functional connectivity. Source data are provided as a Source Data file.

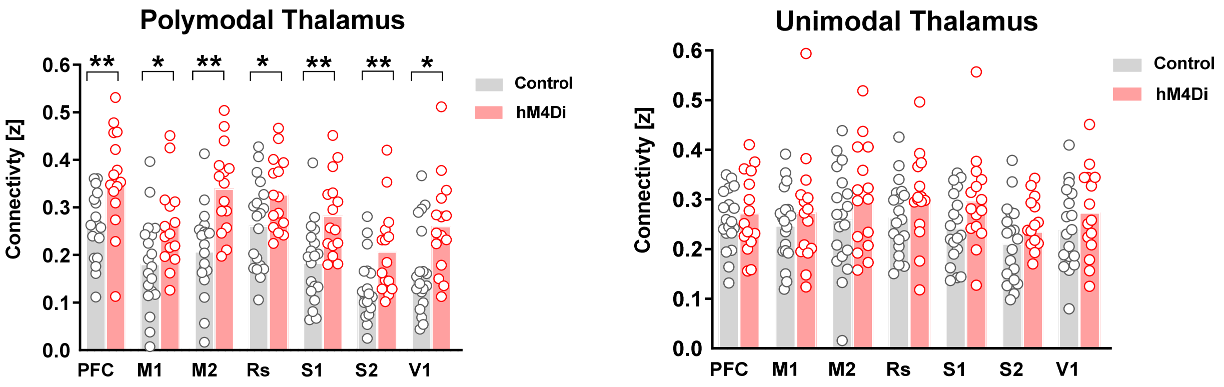

Figure S5. The polymodal thalamus is functionally overconnected to cortical areas. Quantification of thalamo-cortical connectivity extracted from polymodal (left) and unimodal (right) thalamic partitions (*q<0.05, **q<0.01, two-sided t test, FDR corrected, n = 15 and n = 19 hM4Di or GFP-expressing mice, respectively). Source data are provided as a Source Data file. M1: primary motor cortex; M2: secondary motor cortex; S1: primary somatosensory cortex; S1: secondary somatosensory cortex; PFC: prefrontal cortex, RS: retrosplenial cortex; V1: primary visual cortex.

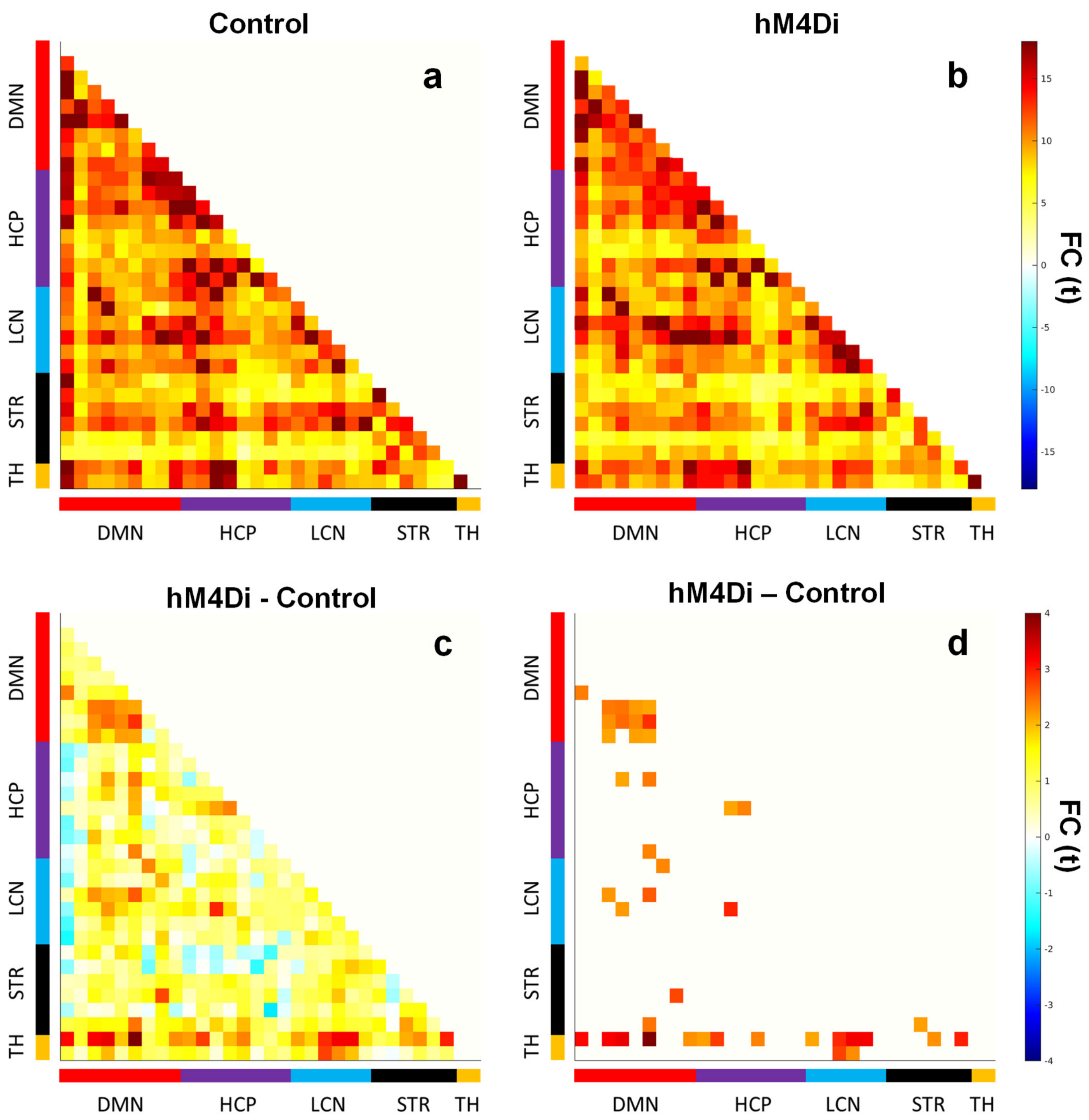

Figure S6. Whole-brain rsfMRI connectivity in control and hM4Di-expressing mice. Correlation matrices in **(a)** and **(b)** depict inter-areal connectivity in control and hM4Di mice respectively. **(c)** Mean difference map (t), and **(d)** regions exhibiting connectivity differences larger than $|t| > 2.1$, corresponding to $p < 0.05$, two-tailed t test (uncorrected). TH: thalamus; STR: striatum; LCN: lateral cortical network; HCP: hippocampus; DMN: default mode network; FC: functional connectivity. Source data are provided as a Source Data file.

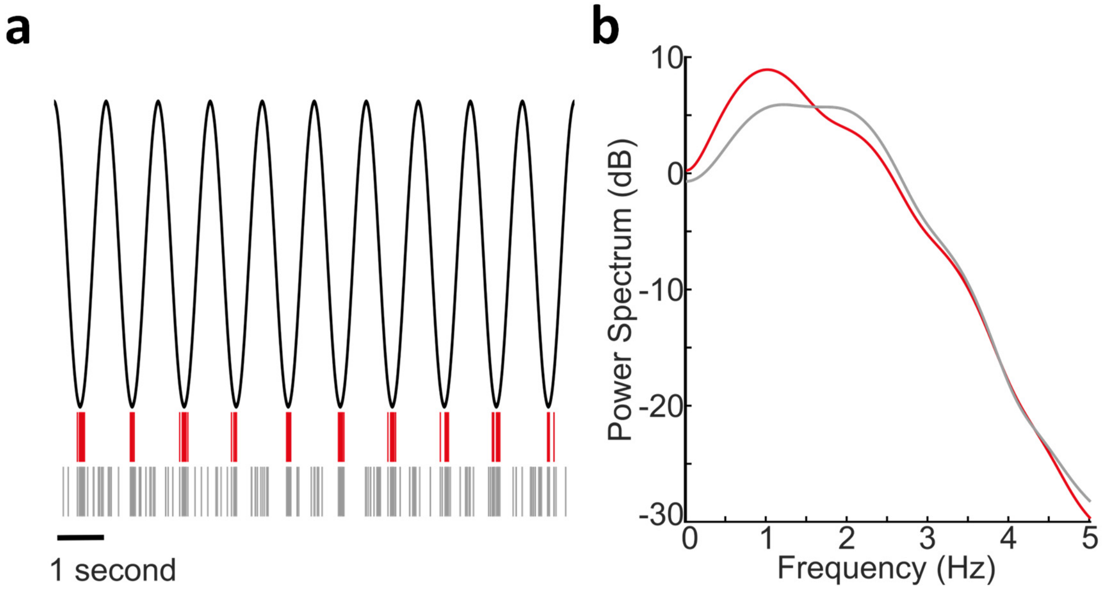

Figure S7. A simple simulation of the effect of suppressing neural firing not locked to slow rhythms. (a) Top (black line): 1 Hz simulated sinusoidal “LFP” wave to which spikes trains are locked. Middle (red lines): spike trains strongly locked to the LFP wave, conceptualizing firing during DREADD-induced manipulation. Bottom (red lines): spike trains obtained from the red ones but adding also an equal number of unlocked spikes, conceptualizing firing during control conditions. (b) Power spectra of the resulting spike trains. Source data are provided as a Source Data file.

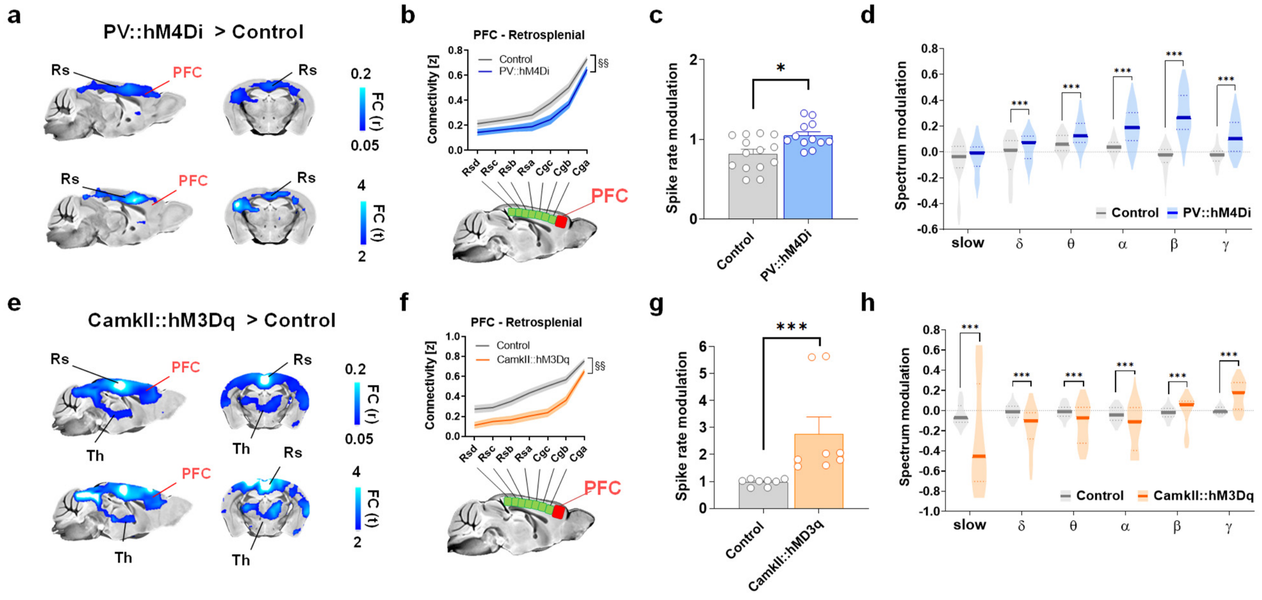

Figure S8. Chemogenetic inhibition of parvalbumin GABAergic cells (a-d) and stimulation of pyramidal neurons (e-h) produce rsfMRI underconnectivity. **(a)** Between group PFC seed-based connectivity difference maps revealed rsfMRI underconnectivity (blue) in the DMN of PV::hM4Di expressing mice during the active CNO phase. PV::Cre mice were bilaterally injected with hSyn-DIO-hM4Di (n = 16). Control PV::Cre animals underwent sham injections (control, n = 17). **(b)** Antero-posterior profiling of rsfMRI connectivity of the PFC along the midline axis of the mouse DMN (§§ p = 0.008, 2-way ANOVA repeated measurements, genotype effect, PV::hM4Di n = 16, sham n = 17). **(c)** Increased firing rate in PV::hM4Di mice upon CNO injection (two-sided Wilcoxon rank-sum test, p = 0.019, n = 12 and n = 14 statistically independent recordings in n = 6 PV::hM4Di and n = 7 control mice, respectively). **(d)** Quantification of the corresponding band-specific power spectrum changes in the PFC (***q < 0.001, two-sided Wilcoxon rank-sum test followed by FDR correction, n = 120 and n = 140 statistically independent recordings in n = 6 PV::hM4Di and n = 7 control mice, respectively). **(e)** Between group PFC seed-based connectivity difference map in mice expressing CamkII::hM3Dq (n = 20), or CamkII::cre mice subjected to sham viral injections (control, n = 17) during the CNO active phase. This analysis revealed the presence of robust rsfMRI underconnectivity (blue) in the DMN. **(f)** Antero-posterior profiling of rsfMRI connectivity of the PFC along the midline axis of the mouse DMN in the two cohorts (§§ p < 0.001, 2-way ANOVA repeated measurements, genotype effect, CamkII::hM3Dq n = 20, sham n = 17). **(g)** Largely increased firing rate in hM3Dq-expressing mice compared to controls (*** p < 0.001, two-sided Wilcoxon rank-sum test, n = 8 and n = 8 statistically independent recordings in n = 4 CamkII::hM3Dq and n = 4 sham mice, respectively). **(h)** Quantification of corresponding band-specific power spectrum changes upon CNO injection (*** q < 0.001, two-sided Wilcoxon rank-sum test followed by FDR correction, n = 80 statistically independent recordings in n = 4 CamkII::hM3Dq and n = 4 control mice, respectively). Data in barplots **(c)** and **(g)** are presented as mean values ± SEM. Violin plots: thick lines represent median, dashed lines indicate 25th and 75th percentile, respectively. Cg: cingulate cortex; PFC: prefrontal cortex, RS: retrosplenial cortex; Th: Thalamus; FC: functional connectivity. Source data are provided as a Source Data file.

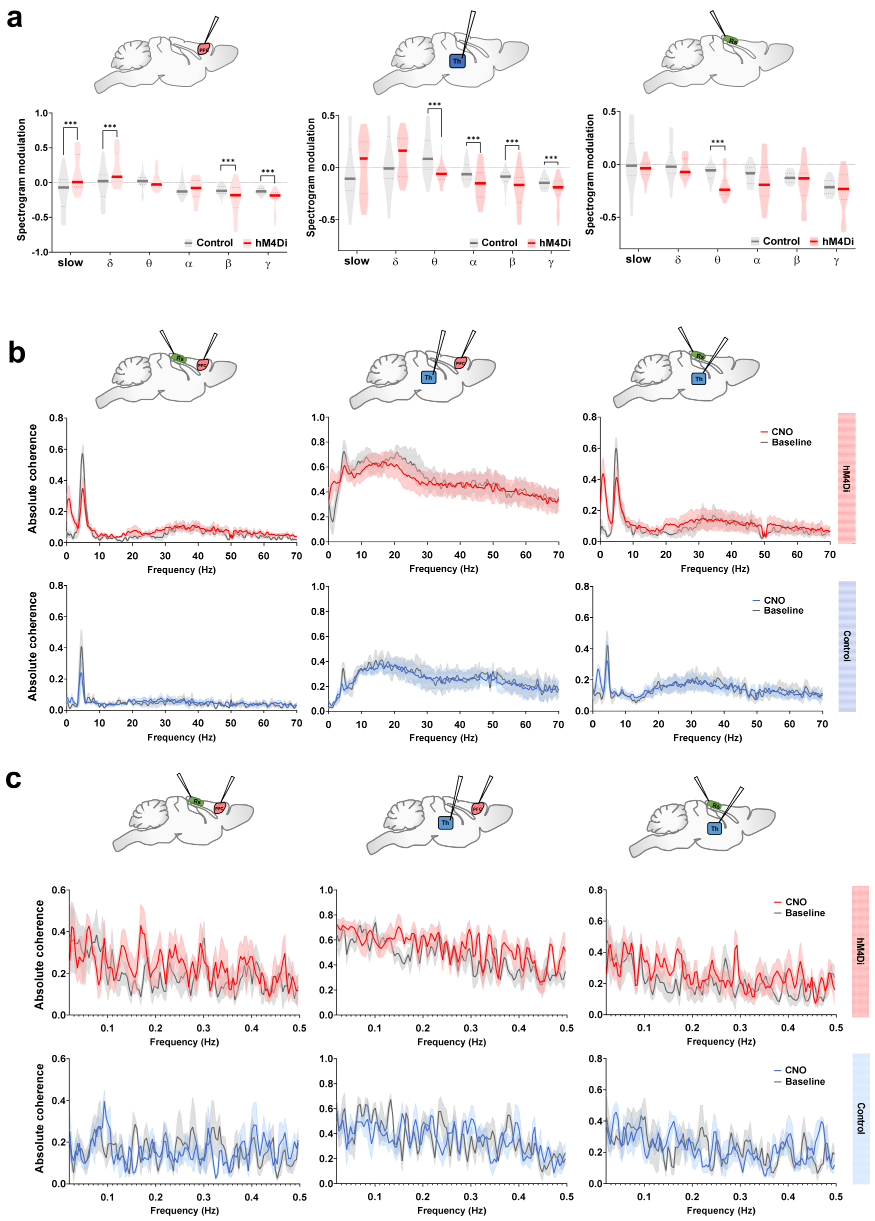

Figure S9. LFP power spectra and absolute coherence in multi-electrode recordings. (a) Quantification of band-specific power spectrum changes in LFPs recorded in the PFC (left), centromedial thalamus (Th; center) and retrosplenial cortex (Rs; right) upon systemic CNO administration. Power was quantified with respect to pre-injection baseline (violin plots: thick lines represent median, dashed lines indicate 25th and 75th percentile, respectively; two-sided Wilcoxon rank-sum tests, followed by FDR correction, ***q<0.001, n = 50 and n = 40 statistically independent recordings in n = 5 hM4Di and n = 4 control mice, respectively). **(b)** Mean absolute power coherence for all pairs of electrophysiologically-probed regions (PFC-Rs; PFC-Th; Rs-Th) during both baseline and CNO active time window in hM4Di and control animals (mean ± SEM). **(c)** Mean absolute power coherence of ultra-slow oscillation for all pairs of electrophysiologically-probed regions (PFC-Rs; PFC-Th; Rs-Th) computed for both baseline and CNO active time window in hM4Di and control animals (mean ± SEM). Source data are provided as a Source Data file.

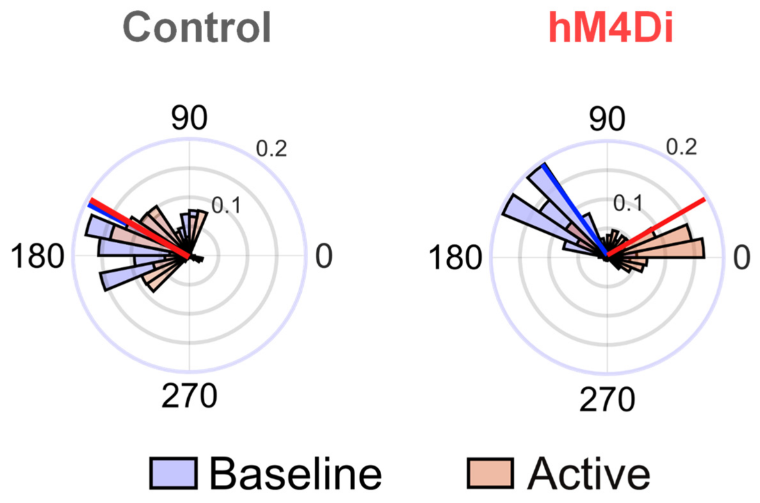

Figure S10. LFP δ-band phase difference between PFC and Rs cortices. Distribution of the difference in average instantaneous phase between PFC and Rs recording channels, before (baseline) and after CNO-administration (active) in control and hM4Di-expressing animals. Thick lines indicate the circular average across channels of the phase difference for baseline (blue) and active (red) phase, respectively. Source data are provided as a Source Data file.

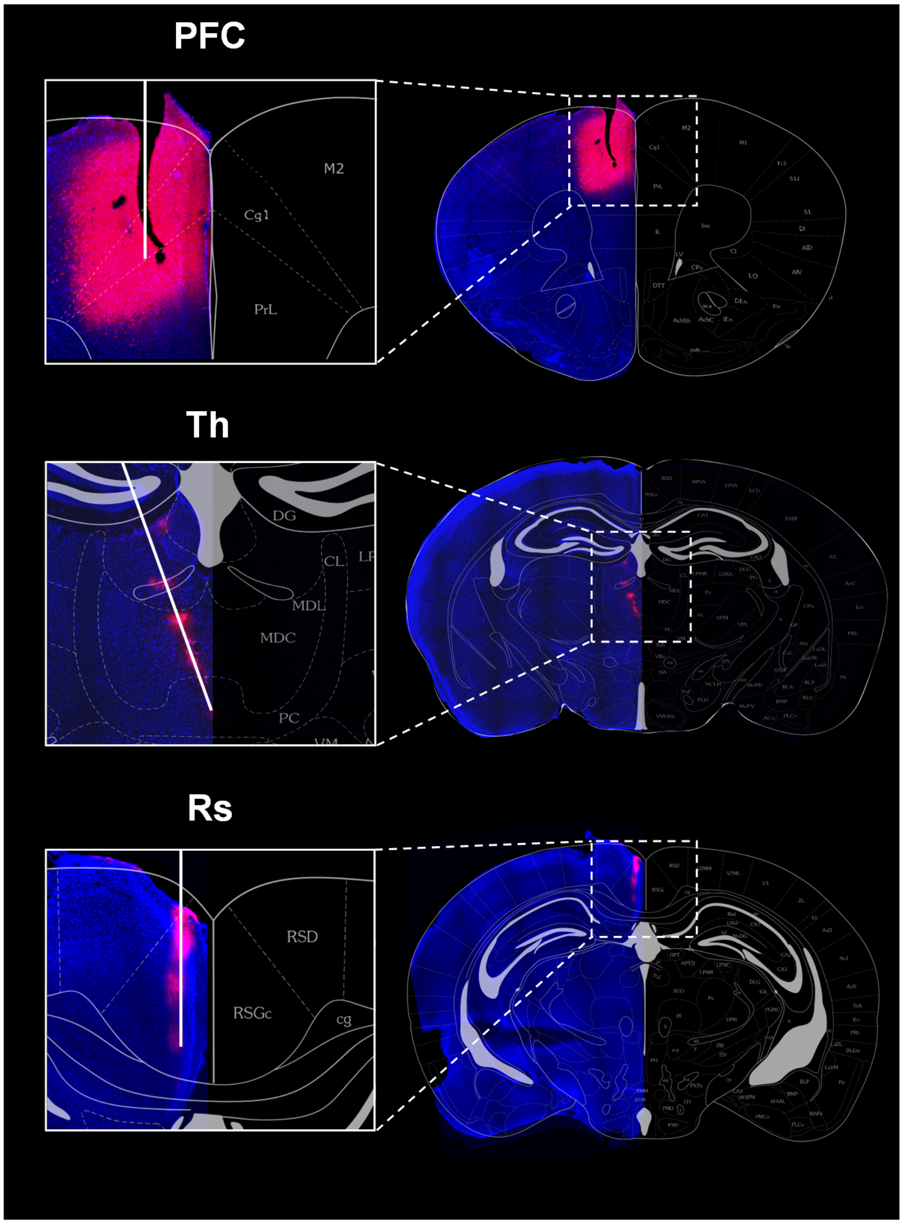

Figure S11. Histological validation of electrode placement. Electrode insertion traces (Th and Rs, red, lipophilic dye DiI) are here highlighted with a white line to indicate approximate electrode profile and insertion angle. In the PFC, red signal represents hSyn-hM4Di viral expression, and area of electrode insertion is indicated by a the corresponding mechanically-induced lesion produced before electrode removal at the end of recording session. Individual color channels have been adjusted to increase contrast. PFC: medial prefrontal cortex; Th: thalamus; Rs: retrosplenial cortex.
