# Supplementary material for: Increased fMRI connectivity upon chemogenetic inhibition of the mouse prefrontal cortex
Source: Nat Commun. 2022 Feb 25;13:1056. doi: 10.1038/s41467-022-28591-3 (PMC8881459; doi:10.1038/s41467-022-28591-3)
Supplement: Supplementary file 2 — Reporting Summary [file 41467_2022_28591_MOESM2_ESM.pdf]

nature research

Corresponding author(s): Alessandro Gozzi

Last updated by author(s): 13/01/2022

Reporting Summary

Nature Research wishes to improve the reproducibility of the work that we publish. This form provides structure for consistency and transparency in reporting. For further information on Nature Research policies, see our Editorial Policies and the Editorial Policy Checklist.

Statistics

For all statistical analyses, confirm that the following items are present in the figure legend, table legend, main text, or Methods section.

n/a	Confirmed	
☐	☒	The exact sample size (n) for each experimental group/condition, given as a discrete number and unit of measurement
☐	☒	A statement on whether measurements were taken from distinct samples or whether the same sample was measured repeatedly
☐	☒	The statistical test(s) used AND whether they are one- or two-sided<br>*Only common tests should be described solely by name; describe more complex techniques in the Methods section.*
☐	☒	A description of all covariates tested
☐	☒	A description of any assumptions or corrections, such as tests of normality and adjustment for multiple comparisons
☐	☒	A full description of the statistical parameters including central tendency (e.g. means) or other basic estimates (e.g. regression coefficient) AND variation (e.g. standard deviation) or associated estimates of uncertainty (e.g. confidence intervals)
☐	☒	For null hypothesis testing, the test statistic (e.g. F, t, r) with confidence intervals, effect sizes, degrees of freedom and P value noted<br>*Give P values as exact values whenever suitable.*
☒	☐	For Bayesian analysis, information on the choice of priors and Markov chain Monte Carlo settings
☐	☒	For hierarchical and complex designs, identification of the appropriate level for tests and full reporting of outcomes
☐	☒	Estimates of effect sizes (e.g. Cohen's d, Pearson's r), indicating how they were calculated

Our web collection on statistics for biologists contains articles on many of the points above.

Software and code

Policy information about availability of computer code

Data collection	RHD Recording Controller software (v2.09) developed by Intan Technologies to acquire electrophysiological data.<br>Paravision (version 6.01) developed by Bruker Biospin to acquire fMRI data.
Data analysis	The code used for preprocessing and analyzing mouse rsfMRI data is available at https://github.com/functional-neuroimaging/rsfMRI-preprocessing, /rsfMRI-global-local-connectivity and /PFC-chemo-inhib.<br>Electrophysiological analyses were carried out using Circstat (v.R2012a), and in house made Matlab code.

For manuscripts utilizing custom algorithms or software that are central to the research but not yet described in published literature, software must be made available to editors and reviewers. We strongly encourage code deposition in a community repository (e.g. GitHub). See the Nature Research guidelines for submitting code & software for further information.

Data

Policy information about availability of data

All manuscripts must include a data availability statement. This statement should provide the following information, where applicable:

- Accession codes, unique identifiers, or web links for publicly available datasets
- A list of figures that have associated raw data
- A description of any restrictions on data availability

The rsfMRI and electrophysiology data generated in this study are under active use by the reporting laboratory; all data presented in this manuscript are available by reasonable request. Source data are provided with this paper (Source_data.xlsx). BOLD parametric maps are available for download at: https://data.mendeley.com/datasets/2b3n86gr25/1

April 2020

Field-specific reporting

Please select the one below that is the best fit for your research. If you are not sure, read the appropriate sections before making your selection.

☒ Life sciences ☐ Behavioural & social sciences ☐ Ecological, evolutionary & environmental sciences

For a reference copy of the document with all sections, see nature.com/documents/nr-reporting-summary-flat.pdf

Life sciences study design

All studies must disclose on these points even when the disclosure is negative.

Sample size

No statistical methods were used to predetermine sample sizes for our experiments, but our sample sizes are comparable or largely exceed those reported in previous publications on mouse rsfMRI by other groups (Zerbi et al., 2021; Grandjean et al., 2020, Liska et al. 2018, Pagani et al. 2019, Bertero et al. 2018, Sutterlin et al. 2018, Sforazzini et al. 2016), or in vivo electrophyisiological recordings upon DREADD modulation in rodents (Rodriguez et al., 2020; Schmidt et al., 2019; Alexander et al., 2018)

Data exclusions

Animals exhibiting unilateral viral expression were removed from rsfMRI and electrophysiological analyses. This exclusion criterion was predetermined.

Replication

The complex and lengthy design of our mouse studies did not allow us to perform formal replication studies for all the employed in vivo readouts, as such endeavor would also have important ethical implications in terms of 3R compliance and animal use. We note however that our main finding (i.e. overconnectivity upon inhibition of the mouse PFC) was replicated with two anesthetics (Fig. 1, 2 and S4) and with two silencing methods (i.e. Kir2.1 overexpression or hM4Di inhibition, Fig. 1 and 2). We also note that electrophysiological evidence of reduced neural firing and increased delta-band power upon DREADD inhibition of the PFC was replicated in two independent studies (Fig. 4 and S9). Collectively, all our attempts at replication were largely successful, and corroborate the robustness of our main findings.

Randomization

Mice were assigned either to the experimental or to the control group by using random number generation as implemented in Matlab

Blinding

All data acquisition and analysis were performed blind to the genotype/treatment and group allocation.

Reporting for specific materials, systems and methods

We require information from authors about some types of materials, experimental systems and methods used in many studies. Here, indicate whether each material, system or method listed is relevant to your study. If you are not sure if a list item applies to your research, read the appropriate section before selecting a response.

Materials & experimental systems

n/a	Involved in the study	
☒	☐	Antibodies
☒	☐	Eukaryotic cell lines
☒	☐	Palaeontology and archaeology
☐	☒	Animals and other organisms
☒	☐	Human research participants
☒	☐	Clinical data
☒	☐	Dual use research of concern

Methods

n/a	Involved in the study	
☒	☐	ChIP-seq
☒	☐	Flow cytometry
☐	☒	MRI-based neuroimaging

Animals and other organisms

Policy information about studies involving animals; ARRIVE guidelines recommended for reporting animal research

Laboratory animals

Kir 2.1 and hSYN/hM4Di inhibition studies, and CamkII-hM3Dq stimulation studies were carried out in adult (6 week old) male C57Bl6/J mice (Jackson laboratories, Stock No: 000664). hM4Di inhibition of parvalbumin-positive neurons was carried out in adult (6 week old) male Parvalbumin-cre mice (Jax. code 017320). Mice were group housed in a 12:12 hour light-dark cycle in individually ventilated cages with access to food and water ad libitum and with temperature maintained at 21 ± 1 °C and humidity at 60 ± 10%.

Wild animals

No wild animals were used in the study.

Field-collected samples

No field collected samples were used in the study.

Ethics oversight

Animal studies were conducted in accordance with the Italian Law (DL 26/2014, EU 63/2010, Ministero della Sanità, Roma) and the recommendations in the Guide for the Care and Use of Laboratory Animals of the National Institutes of Health. Animal research

protocols were reviewed and consented to by the animal care committee of the Istituto Italiano di Tecnologia and the Italian Ministry of Health (852/17 to A.G). All surgical procedures were performed under anesthesia.

Note that full information on the approval of the study protocol must also be provided in the manuscript.

Magnetic resonance imaging

Experimental design

Design type	Resting state fMRI acquisition
Design specifications	Single-shot BOLD rsfMRI time series were acquired using an EPI sequence
Behavioral performance measures	none

Acquisition

Imaging type(s)	fMRI
Field strength	7T
Sequence & imaging parameters	TR/TE 1000/15 ms, flip angle 60°, matrix 98 x 98, FOV 2.3 x 2.3 cm, 18 coronal slices, slice thickness 550 μm
Area of acquisition	whole brain scans

Diffusion MRI ☐ Used ☒ Not used

Preprocessing

Preprocessing software	Image time series data were pre-processed with tools from functional magnetic resonance imaging of the brain (Oxford) (FMRIB) Software Library (FSL, v.4.1.4; http://www.fmrib.ox.ac.uk/fsl), analysis of functional neuroimages (AFNI, v.2011_12_21_1014; http://afni.nimh.nih.gov) software and MATLAB (Math-Works)
Normalization	Linear normalization to group standardized space using FSL's FLIRT
Normalization template	In-house EPI mouse brain template available at Coletta et al., Sci Adv 2020
Noise and artifact removal	After registration, all the functional images were realigned (correction for motion) and the six head motion traces were regressed from the time series data. In order to minimise non-neural signal contributions, the mean ventricular signal (averaged fMRI time course within a manually-drawn ventricle mask) was considered as nuisance signal and regressed out of each of the time series for each subject
Volume censoring	none

Statistical modeling & inference

Model type and settings	none
Effect(s) tested	none

Specify type of analysis: ☐ Whole brain ☐ ROI-based ☒ Both

Anatomical location(s)	Our anatomical definition reflects recent neuroanatomical and cytoarchitectural cross-species comparisons
Statistic type for inference (See Eklund et al. 2016)	voxel-wise
Correction	FDR and cluster correction

Models & analysis

n/a	Involved in the study
☐	☒ Functional and/or effective connectivity
☒	☐ Graph analysis
☒	☐ Multivariate modeling or predictive analysis

Functional and/or effective connectivity	Pearson correlation
